# Sensor-Uncertainty-Aware Conservative Robust Route Selection for Autonomous Robot Path Planning Under Occupancy-Grid Map Uncertainty

**DOI:** 10.3390/s26165172

**Published:** 2026-08-15

**Authors:** Ali S. Allahloh, Atef M. Ghaleb, Mohammad Sarfraz, Abdelghani Bouras, Mohammed A. H. Ali, Adel Al-Shayea

**Affiliations:** 1 Department of Electrical Engineering, Zakir Husain College of Engineering and Technology (ZHCET), Aligarh Muslim University, Aligarh 202002, India; msarfraz@zhcet.ac.in; 2 Department of Industrial Engineering, College of Engineering and Advanced Computing, Alfaisal University, Riyadh 11533, Saudi Arabia; 3 Department of Mechanical Engineering, Faculty of Engineering, Universiti Malaya, Kuala Lumpur 50603, Malaysia; 4 Industrial Engineering Department, College of Engineering, King Saud University, Riyadh 11421, Saudi Arabia

**Keywords:** robotic path planning, sensor uncertainty, autonomous navigation, robust optimization, route selection, collision avoidance, occupancy grids, scenario-based planning, dynamic environments, CVaR

## Abstract

Autonomous robotic navigation in dynamic environments depends on sensor-derived occupancy maps that are often degraded by occlusion, localization error, dynamic blockage, incomplete observation, and perception noise. These uncertainties can make a nominally short route unsafe after deployment, motivating conservative route selection for collision-aware path planning under sensor-derived map uncertainty. We formulate Conservative Robust Route Selection (CRRS) as a finite-scenario robust optimization and route-selection framework for autonomous robotic path planning under this uncertainty. CRRS constructs a heterogeneous portfolio of candidate routes, scores each route using nominal and plausible-world information only, and applies a validation-frozen conservative override rule that defaults to the scenario ensemble unless a candidate route satisfies predefined feasibility, risk, clearance, and cost-ratio guards. The evaluation protocol separates implementation auditing, candidate-pool expansion, validation-based selector design, frozen confirmation, public-benchmark validation, simulated sensor-model validation, and a controlled validation–held-out mismatch stress test. On the generated 30-domain MovingAI-format benchmark, candidate-pool expansion finds strict-safe-superior candidates in 113/150 matched groups, and the frozen selector reduces plausible collision from 0.1250 to 0.0807 and held-out collision from 0.3053 to 0.2937. On an official long-distance MovingAI subset with 260 queries and a minimum start-goal distance of 100 cells, CRRS reduces the held-out collision from 0.9648 for the scenario ensemble to 0.8822, with 141 wins, zero losses, and 119 ties. In an additional LiDAR/SLAM-inspired simulated sensor-model validation on 200 routed official-query problems, CRRS reduces the held-out collision from 0.4059 to 0.3768 relative to the scenario ensemble. A validation–held-out mismatch stress ablation isolates the conservative override rule: CRRS differs from CVaR-only on 73/260 problems, obtains a lower or equal held-out collision in every comparison, and avoids the 18 harmful held-out losses incurred by CVaR-only relative to the scenario ensemble. The resulting claim is deliberately scoped: CRRS improves aggregate route robustness over a strong scenario-ensemble default on the evaluated robotic path-planning benchmarks, while real-time deployment, live sensor integration with calibrated sensors, physical robot validation, family-level variation, and benchmark-specific uncertainty models remain limitations.

## 1. Introduction

Autonomous mobile robots increasingly rely on occupancy grids, cost maps, and traversability maps generated from onboard or external sensing. These maps are often treated as fixed planning inputs, although in realistic deployments they are affected by occlusion, localization error, perception noise, map aging, dynamic blockage, and incomplete observation. A route that is optimal on the nominal map may therefore commit the robot to a corridor that becomes unsafe or infeasible under plausible sensor-derived realizations of the environment. This is especially problematic in dynamic or partially observed environments, where corridor-level uncertainty can matter more than small local path-length differences.

Classical path planners often commit early to a single corridor that is optimal in the available map. This is appropriate when the map is accurate and the environment is static, but it is fragile when occupancy, predicted blockage, or clearance changes across plausible realizations of the environment. A nominally short path may pass through a passage that is frequently obstructed under realistic perturbations, while a slightly longer route may generalize better across worlds. The central question addressed here is therefore a robotic route-selection question under sensor-derived environment uncertainty: how can a planner preserve multiple route commitments long enough to select the route that offers the best finite-scenario robustness under a fixed information boundary?

This paper studies that question through Conservative Robust Route Selection (CRRS), a grid-based robust optimization framework for autonomous robotic path planning. CRRS first constructs a robust clearance substrate from plausible worlds, then builds a compact candidate portfolio from heterogeneous planning sources, and finally applies a validation-frozen conservative override rule before optional guarded shortcut refinement. The method can be viewed as a finite-scenario, source-aware portfolio selector: it optimizes over completed route candidates rather than over individual grid edges alone, and it makes the conservative scenario-ensemble route the default action. The design is intentionally limited in scope: it does not solve belief-space planning, does not provide a distribution-free safety guarantee, does not perform low-level robot control, and does not claim real-time superiority over lightweight graph search. Its claim is more specific: when sensor-derived environment uncertainty can invalidate the nominal corridor, a route portfolio scored on planning-time plausible worlds can reduce collision relative to the scenario ensemble and single-route nominal or robust shortest-path baselines.

Terminology

The term “conservative” is used deliberately. CRRS is not a causal-inference method and does not rely on structural causal models, interventions, or causal counterfactual queries. The method is a finite-scenario robust route selector over sampled or generated plausible occupancy-grid worlds. The conservative property refers to the validation-frozen rule that defaults to the scenario ensemble unless an alternative candidate satisfies predefined risk, feasibility, clearance, and cost-ratio guards.

Special Issue relevance

This study is aligned with the Special Issue focus on advanced control and path-planning techniques for autonomous robotic systems in dynamic environments. CRRS addresses the route-commitment decision that occurs after a robot has constructed sensor-derived occupancy or traversability maps but before low-level tracking, local reactive avoidance, or actuator-level control is applied. By preserving route alternatives and selecting among them using plausible collision, feasibility, cost, and clearance evidence, the framework provides robust navigation and control/path-planning decision support for autonomous robots operating under dynamic or partially observed map uncertainty. The present work therefore targets autonomous path planning and collision-aware route selection; it does not claim collaborative multi-robot coordination.

Table 1 separates the evidence demonstrated in this paper from the downstream robotic validation that remains future work. This table is included to keep the title, abstract, contribution claims, and limitations aligned with the actual evidence.

The contributions are as follows:

1. A sensor-uncertainty-aware finite-scenario route-selection formulation for occupancy-grid robot navigation under a strict nominal/plausible/held-out information boundary.
2. A heterogeneous route-portfolio construction strategy that preserves alternatives from scenario, robust-substrate, graph-search, PRM/homotopy, and visibility-style sources where supported by the experiment package.
3. A validation-frozen conservative override selector that defaults to the scenario ensemble unless risk, feasibility, clearance, and cost-ratio guards admit an alternative route.
4. A staged non-oracle evaluation protocol separating candidate-pool headroom, selector design, frozen generated-benchmark confirmation, official MovingAI validation, simulated LiDAR/SLAM-inspired sensor-model validation, and validation–held-out mismatch stress ablation.
5. Reproducible benchmark evidence showing aggregate route-level robustness improvement over a strong scenario-ensemble default under the stated information boundary, while explicitly limiting claims about physical deployment, real-time control, and uniform per-family dominance.

**Figure 1:**
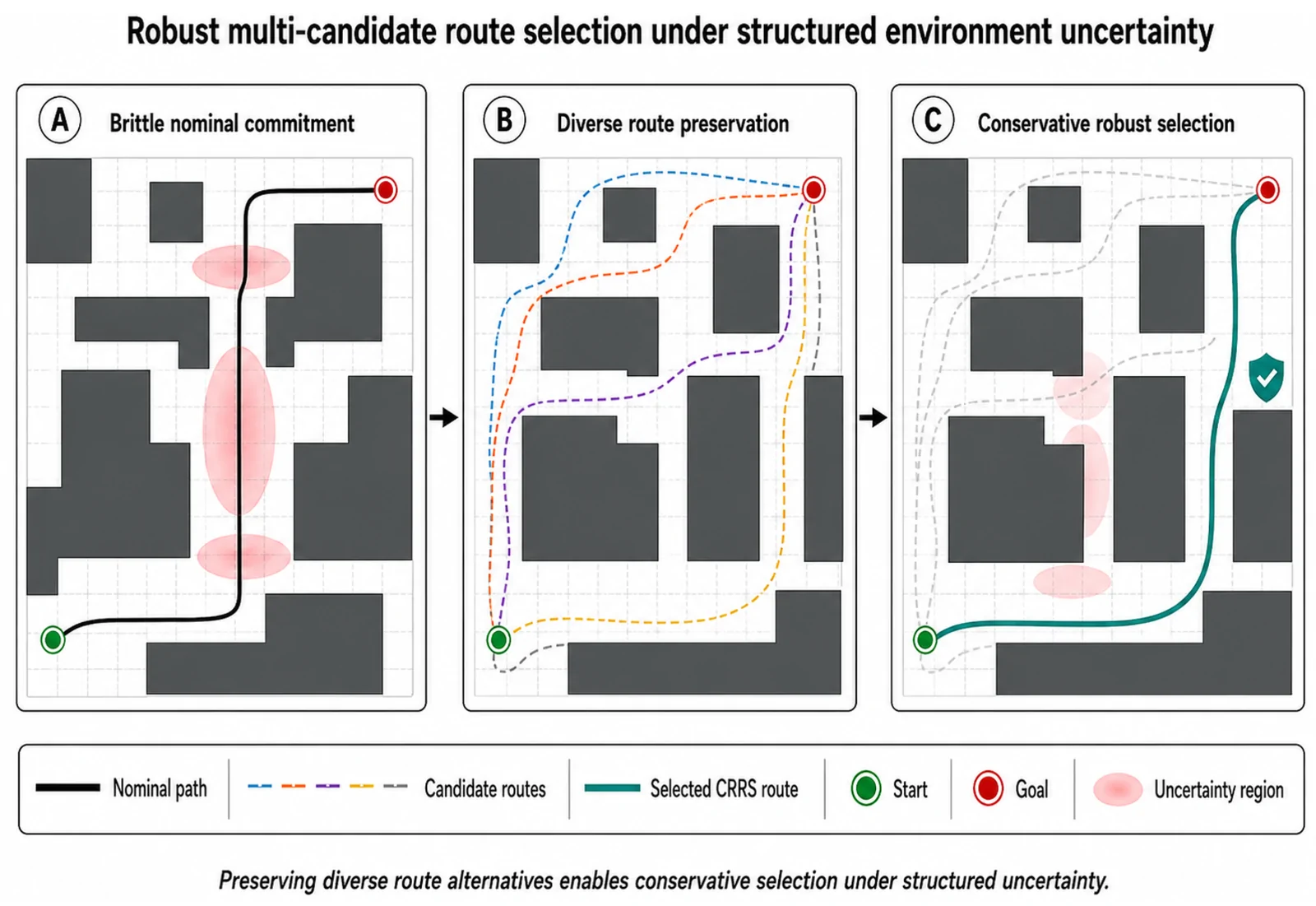
Robust multi-candidate route selection under structured environment uncertainty. A nominal shortest path can commit to a brittle corridor when plausible environmental changes affect traversability. CRRS preserves diverse route alternatives and selects a safer route using nominal and plausible-world evidence. The figure is a conceptual teaser and does not report benchmark results.

Figure 1 illustrates the motivation for preserving multiple route candidates under structured environment uncertainty.

## 2. Related Work

Graph-search planners such as A* remain core tools for grid and roadmap planning because of their simplicity, heuristic structure, and predictable behavior [1]. Replanning and anytime variants, including D* Lite and ARA*, extend this family to changing costs or bounded-suboptimal operation [2,3]. Any-angle methods such as Theta* reduce grid-induced path artifacts by allowing line-of-sight shortcuts during graph search [4]. These methods are strong baselines, but their standard form still returns a single route under a selected map or cost field.

Occupancy-grid mapping and probabilistic mobile-robot localization motivate the uncertainty interface used here. Classical occupancy grids model spatial evidence accumulated from range sensing, while SLAM and particle-filter localization expose uncertainty caused by pose error, noisy observations, map aging, and incomplete observation [5,6,7,8]. In navigation stacks, these uncertainty sources are often converted into cost-map inflation, traversability scores, or alternative map hypotheses before global planning. CRRS operates at that route-selection interface: it does not estimate the map or solve SLAM, but it consumes a nominal occupancy/traversability grid together with plausible alternatives generated by the perception, mapping, prediction, or Digital Twin layer.

Sampling-based planners and roadmap methods address different aspects of the planning problem. Probabilistic roadmaps and RRT* provide scalable planning tools with probabilistic-completeness or asymptotic-optimality properties under their respective assumptions [9,10,11]. Optimization-based planners such as CHOMP improve continuous trajectories once an initial route is available [12]. These approaches can be combined with robust costs, but they do not by themselves resolve the route-commitment problem when uncertainty makes one homotopy class safer than another.

Diverse path generation is closely related. Classical k-shortest-loopless-path algorithms explicitly enumerate alternatives [13], and homotopy- or topology-aware planners aim to distinguish route classes rather than merely return small geometric perturbations of the same path [14,15]. CRRS uses this perspective operationally: route diversity is valuable because uncertainty can make an apparently inferior corridor safer under plausible perturbations.

Planning under uncertainty includes chance-constrained planning, risk-aware optimization, and scenario-based motion planning [16,17,18]. Scenario-based methods are particularly relevant because they optimize or test decisions across sampled realizations of uncertainty. CRRS differs from a pure scenario ensemble by separating candidate generation from final candidate selection: the final planner can use scenario-based, robust-substrate, nominal, and visibility candidates, but the selector compares complete routes with the same fixed risk score.

Reactive local planners and learning-based navigation policies address complementary levels of the robotic navigation stack. Methods such as the Dynamic Window Approach [19] select admissible velocity commands from local obstacle and goal information, while deep reinforcement learning policies [20] can learn reactive behaviors that adapt to cluttered or partially observed environments. These methods are valuable for local collision avoidance and closed-loop execution, but they do not by themselves resolve the upstream route-commitment problem that arises when different corridors have different robustness under sensor-derived map uncertainty. CRRS is positioned at this route-selection level: it preserves multiple completed route alternatives, evaluates them across plausible occupancy-grid realizations, and selects a conservative route before low-level tracking or reactive control is applied.

Skeleton- and roadmap-based methods provide sparse connectivity representations of free space. Generalized Voronoi graphs are a classical clearance-aware construction [21]. The present work follows the same motivation but uses robust clearance quantiles over plausible worlds to build a substrate from which alternative route commitments can be proposed and compared.

The experimental protocol is also influenced by motion-planning benchmarking practice, including OMPL and related reproducibility infrastructures [22,23,24]. The present study remains simulation-based and grid-based, but it provides fixed configurations, exported result tables, plotting scripts, and per-run outputs to make the evidence path reproducible.

Taken together, this literature motivates the route-selection role of CRRS. The proposed method is not intended to replace A*, Theta*, PRM, or visibility-roadmap construction, scenario-based planning, reactive local control, reinforcement-learning navigation policies, or trajectory optimization. Instead, CRRS treats these methods and planning concepts as candidate sources, comparators, or complementary components in a robotic navigation stack. Its contribution is a conservative route-level selector over heterogeneous candidate routes under sensor-derived map uncertainty: completed routes are evaluated using nominal and plausible-world evidence, filtered by admissibility and dominance guards, and allowed to override the scenario-ensemble default only when the frozen rule admits the candidate. Held-out worlds remain excluded from candidate construction, scoring, filtering, selector design, inference, and guarded refinement, so the resulting claim is benchmark-scoped route-robustness evidence rather than a distribution-free safety guarantee, a real-time superiority claim, or a hardware-deployment claim.

## 3. Method

Table 2 fixes the terminology used throughout the paper. The distinction between plausible worlds and held-out worlds is central: deployable CRRS operations may use the nominal map and planning-time plausible worlds, whereas held-out worlds are reserved for post-selection evaluation only.

### 3.1. Problem Formulation and Information Boundary

Let $W_{0}$ be the nominal binary occupancy grid available to the robot at planning time, constructed from the current map or sensor-derived traversability estimate, with start *s* and goal *g*. Let $W_{pl}={\left\{W_{i}\right\}}_{i=1}^{m}$ denote plausible occupancy-grid realizations available during planning. In the intended robotic-navigation interpretation, these plausible worlds represent sampled map uncertainty induced by sensing and perception effects such as occlusion, localization error, map aging, dynamic blockage, incomplete observation, or uncertainty in traversability prediction. Let $W_{ho}={\left\{{\tilde{W}}_{j}\right\}}_{j=1}^{h}$ denote held-out worlds used only for evaluation. The planner returns a path $\pi =(x_{1},\ldots ,x_{n})$ or no path. A returned path is rasterized by line interpolation between consecutive waypoints, and collision is tested at the resulting grid samples.

For a path π and world *W*, define $I_{W}\left(\pi \right)=1$ if the path collides with an obstacle or leaves the grid, and $I_{W}\left(\pi \right)=0$ otherwise. Path length is Euclidean over consecutive waypoints. If a path is collision-free in *W*, its cost is

(1) $$C_{W}\left(\pi \right)=L\left(\pi \right)+\frac{1}{\max(d_{W}^{min}\left(\pi \right),10^{-3})},$$

where $d_{W}^{min}\left(\pi \right)$ is the minimum distance-transform clearance along the rasterized path. If the path collides or is missing, $C_{W}\left(\pi \right)$ is set to the infeasible penalty $M=10^{6}$. Held-out quantities $I_{{\tilde{W}}_{j}}$ and $C_{{\tilde{W}}_{j}}$ are never used by CRRS during route construction, filtering, or selection.

### 3.2. Sensor-Derived Uncertainty Interface and Benchmark Perturbation Interpretation

The CRRS abstraction separates the sensing-derived planning state from the evaluation-only uncertainty state. The nominal world $W_{0}$ denotes the occupancy or traversability grid available at planning time, produced from the current sensor, mapping, or traversability-estimation pipeline. The plausible-world set $W_{pl}$ contains planning-time alternative occupancy or traversability realizations that CRRS may use for candidate generation, route scoring, dominance filtering, conservative override admission, inference, and guarded refinement. The held-out set $W_{ho}$ is reserved strictly for post-selection evaluation: no held-out collision, cost, clearance, or feasibility quantity is used during candidate construction, scoring, filtering, selector design, inference, or shortcut refinement.

In a deployed robotic sensing pipeline, plausible worlds could be generated from LiDAR occupancy uncertainty, stereo-vision depth uncertainty, sonar or underwater perception uncertainty, SLAM pose and covariance uncertainty, occupancy-grid aging or map staleness, temporary blockage and dynamic obstacles, and traversability-prediction confidence from learned perception models. Table 3 maps the benchmark perturbation interface to common robotic sensing and mapping phenomena. The mapping is interpretive rather than a claim of calibrated sensor simulation: the benchmark does not reproduce the physics, timing, covariance propagation, or perception stack of a specific LiDAR, camera, sonar, or multi-sensor SLAM system. Instead, it provides controlled and repeatable occupancy-grid uncertainty surrogates for isolating the route-selection layer.

This distinction is important for the claim. CRRS addresses the decision layer that consumes a nominal map and plausible alternative worlds from perception, mapping, prediction, Digital Twin, or hardware-in-the-loop infrastructure. Physical sensor calibration, live SLAM/perception integration, ROS/Nav2 or Gazebo execution, closed-loop tracking, and hardware trials remain outside the evidence supplied here and are listed as future validation steps rather than implied deployment results.

### 3.3. Finite-Scenario Robust Route-Selection View

Let $\Pi =\{\pi_{1},\ldots ,\pi_{K}\}$ be the candidate route portfolio generated before final selection, and let D(Π) denote the subset that survives dominance, nominal-success, feasible-fraction, admissible-source, and cost-ratio guards. CRRS is then a constrained finite-scenario selector over complete routes,

(2) $$\pi^{⋆}=\underset{\pi \in D(\Pi )}{\mathrm{arg min}}\;\Phi \left(\pi ;W_{0},W_{pl}\right),$$

where Φ is the lexicographic risk–feasibility–cost–clearance order defined below, with the scalar score in Equation (10) used only as a late tie-breaker. If no admissible override satisfies the frozen validation rule, the selector returns the scenario-ensemble route $\pi_{s}$. This formulation is important for reproducibility and for avoiding leakage: every term in Φ is a function of $W_{0}$ and $W_{pl}$ only, whereas $W_{ho}$ is reserved for post-selection evaluation.

### 3.4. Robust Clearance Substrate

For each plausible world $W_{i}$, let $d_{i}\left(x\right)$ be the Euclidean distance-transform clearance at grid cell *x*. The robust clearance field is a lower quantile over plausible worlds,

(3) $$\rho_{\alpha_{g}}\left(x\right)={\mathrm{Quantile}}_{\alpha_{g}}\left(d_{1}\left(x\right),\ldots ,d_{m}\left(x\right)\right),$$

with robust free set

(4) $$F_{\rho }=\left\{x:\rho_{\alpha_{g}}\left(x\right)\geq \rho_{min}\right\}.$$

The start and goal cells are retained even when local uncertainty is severe, so that failure is expressed through candidate invalidity or high risk rather than by trivially deleting the endpoints. A clearance-weighted grid cost,

(5) $$G\left(x\right)=1+w_{c}\;\frac{1}{\max(\rho_{\alpha_{g}}\left(x\right),10^{-3})},$$

is used by robust A*/Theta* and by portfolio candidates generated on the robust substrate.

### 3.5. Candidate Portfolio

CRRS is best viewed as a conservative portfolio selector. The candidate pool contains paths from: (i) robust-substrate generation, (ii) scenario ensemble, (iii) nominal A*, (iv) robust A*, (v) robust Theta*, (vi) nominal visibility-roadmap planning, and (vii) robust visibility-roadmap planning. Invalid candidates are retained only as high-cost options and cannot win unless all feasible alternatives fail.

This design affects how comparisons should be interpreted. Since CRRS may include scenario-ensemble and A*/Theta*/visibility candidates in its portfolio, the method is not claimed to be a purely independent replacement for those planners. Instead, it is evaluated as a source-aware selector that decides when to use a robust-substrate route, a scenario route, or a simpler graph-search route under the same plausible-world scoring rule.

### 3.6. Candidate Features and Dominance Filter

For each candidate π, define the plausible collision rate,

(6) $${\hat{p}}_{pl}\left(\pi \right)=\frac{1}{m}\sum_{i=1}^{m}I_{W_{i}}\left(\pi \right),$$

and the blocked-world rate,

(7) $$b_{pl}\left(\pi \right)=\frac{1}{m}\sum_{i=1}^{m}1\left\{C_{W_{i}}\left(\pi \right)\geq 0.99M\right\}.$$

The plausible-world tail cost is computed with the upper-tail conditional value at risk,

(8) $${\mathrm{CVaR}}_{\beta }\left(C_{i}\left(\pi \right)\right)=\frac{1}{|T_{\beta }|}\sum_{i\in T_{\beta }}C_{W_{i}}\left(\pi \right),\;T_{\beta }=\left\{i:C_{W_{i}}\left(\pi \right)\geq Q_{\beta }\left({\left\{C_{W_{k}}\left(\pi \right)\right\}}_{k=1}^{m}\right)\right\}.$$

Robust-substrate geometric features include the mean robust clearance $\bar{\rho }\left(\pi \right)$, the 10th-percentile robust clearance $q_{0.10}^{\rho }\left(\pi \right)$, the robust violation rate $v_{\rho }\left(\pi \right)$, and the outside-robust-free-set rate $o_{\rho }\left(\pi \right)$ measured over rasterized path samples.

The dominance filter first identifies the best plausible collision rate $p^{*}$ and best robust violation rate $v^{*}$ among finite-score candidates. A candidate survives if

(9) $${\hat{p}}_{pl}\left(\pi \right)\leq p^{*}+\epsilon_{p},\;v_{\rho }\left(\pi \right)\leq v^{*}+\epsilon_{v},$$

with default values $\epsilon_{p}=0.03$ and $\epsilon_{v}=0.08$. If no candidate satisfies both conditions, the filter falls back to the plausible-collision condition alone.

### 3.7. Base Route-Selection Score

The final scalar score used inside the surviving candidate set is

(10) $$\begin{array}{cc} S(\pi )= & \;85{\hat{p}}_{pl}\left(\pi \right)+20b_{pl}\left(\pi \right)+2v_{\rho }\left(\pi \right)+0.75\Delta_{q10}\left(\pi \right)+0.40o_{\rho }\left(\pi \right)\\ \; & +0.003{\mathrm{CVaR}}_{\beta }\left(C_{i}\left(\pi \right)\right)+0.001{\bar{C}}_{pl}\left(\pi \right)+0.012C_{0}\left(\pi \right)\\ \; & -0.060d_{0}^{min}\left(\pi \right)-0.035\bar{\rho }\left(\pi \right)-0.015{\bar{d}}_{pl}\left(\pi \right), \end{array}$$

where $\Delta_{q10}\left(\pi \right)=\max\left(\rho_{min}-q_{0.10}^{\rho }\left(\pi \right),0\right)$, ${\bar{C}}_{pl}$ is the mean plausible-world cost, $C_{0}$ and $d_{0}^{min}$ are the nominal cost and nominal minimum clearance, and ${\bar{d}}_{pl}$ is the mean plausible-world minimum clearance. Lower scores are better. Table 4 lists the terms and weights.

Candidate choice is lexicographic before using the scalar score as a late tie-breaker. Surviving candidates are sorted by

(11) $$\left({\hat{p}}_{pl},I_{W_{0}},b_{pl},{\mathrm{CVaR}}_{\beta },C_{0},v_{\rho },o_{\rho },S,r_{source}\right),$$

where $r_{source}$ is a deterministic source rank used only to make ties reproducible. This ordering makes collision and feasibility dominate small cost differences. Crucially, no held-out collision, held-out cost, or held-out clearance term appears in either the dominance filter, scalar score, or lexicographic order.

### 3.8. Weight Hierarchy and Selector Sensitivity Rationale

The weights in Equation (10) should be interpreted within the full selector hierarchy rather than as an unconstrained black-box weighted sum. Candidate admissibility guards and the validation-frozen conservative override rule are applied before any deployable override is accepted. Within the surviving candidate set, the lexicographic order in Equation (11) prioritizes plausible collision, nominal success, blocked-world rate, tail risk, nominal cost, and robust-substrate violation before the scalar score is used as a late tie-breaker. The large coefficients on plausible collision and infeasibility terms intentionally make safety-related quantities dominate smaller cost and clearance preferences; the cost and clearance terms mainly resolve tradeoffs among candidates with similar collision and feasibility behavior. All weights, guards, and tie-breakers are functions of $W_{0}$ and $W_{pl}$ only in the final deployable selector. No held-out collision, cost, clearance, or feasibility metric is used for scoring, thresholding, final-confirmation tuning, inference, or guarded refinement. Section 5.9 reports a compact parameter-sensitivity audit over the main quantile, tail-risk, and clearance settings; it should be interpreted as a local robustness check, not an exhaustive global hyperparameter proof.

### 3.9. Validation-Frozen Conservative Override Selector

CRRS adds a final selector layer to avoid the failure mode observed in direct heuristic overrides: a route can look safer on planning-time worlds while worsening held-out collision. The candidate-pool expansion step therefore enlarges the raw candidate pool before any selector tuning. The expanded pool includes baseline scenario and robust-substrate candidates, higher-scenario-count candidates, scenario-clearance hybrids, widened-uncertainty candidates, PRM homotopy candidates, visibility candidates, and clearance-max nominal candidates. This expansion is used first as an oracle headroom audit only; oracle variants can inspect held-out outcomes and are therefore not deployable selectors.

The validation-based selector converts that headroom into a non-oracle rule. The selector defaults to the scenario ensemble and overrides only when a validation-trained rule admits a candidate using planning-time features. The frozen admissible-source set used in the final confirmation is

(12) $$G_{\mathrm{CRRS}}=\left\{\text{higher-scenario-count},\;\mathrm{PRM}\;\mathrm{homotopy}\;\mathrm{diversity}\right\},$$

with a minimum plausible advantage of 0.02, a maximum candidate plausible collision of 0.30, a minimum plausible feasible fraction of 0.75, a maximum cost ratio to the scenario ensemble of 1.40, and required nominal success. Among candidates passing the rule, the tie-breaking order is minimum plausible collision, maximum plausible advantage, lower cost ratio, and higher clearance. Held-out collision is not available to the selector at inference time.

The frozen-confirmation stage applies this CRRS rule to the final 30-domain benchmark without grid search, retuning, or threshold adjustment. This final stage is the only stage allowed to support the benchmark-scoped superiority claim. Oracle headroom results are reported to explain candidate-pool capacity, not to claim deployable superiority.

### 3.10. Selector-Level Fallback Property

Let $R_{v}\left(\pi \right)$ denote the validation surrogate risk used by the selector, let $\pi_{s}$ be the scenario-ensemble default, and let Γ be the frozen admissible override set.

**Proposition** **1** (Validation-surrogate fallback)**.** *Assume the deployed selector returns $\pi_{s}$ unless there exists π∈Γ satisfying the frozen feasibility, clearance, and cost-ratio guards and*

(13) $$R_{v}\left(\pi \right)\leq R_{v}\left(\pi_{s}\right)-\delta ,$$

*for a fixed margin δ>0. Then, every accepted override improves the scenario-ensemble default by at least δ under $R_{v}$, and every abstention returns $\pi_{s}$ exactly.*

**Proof.**  If the selector accepts an override π, the admission rule requires Equation (13); hence $R_{v}\left(\pi_{s}\right)-R_{v}\left(\pi \right)\geq \delta$. If no candidate satisfies the guards and margin condition, the algorithm definition returns the default $\pi_{s}$. Therefore, the stated fallback property follows directly from the frozen selector rule.    □

This proposition is intentionally modest: it is a selector-level property under the validation surrogate actually used by the selector, not a distribution-free safety guarantee. The stress ablation in Section 5.7 tests whether this abstaining rule prevents harmful low-CVaR overrides when validation evidence underestimates held-out brittleness.

### 3.11. Algorithm Summary and Computational Scaling

Algorithm 1 summarizes the planning pipeline. For a grid with *N* cells, *m* plausible worlds, *K* retained candidates, and P(π) raster samples along path π, distance-transform construction scales as O(mN), candidate scoring scales as $O(\sum_{\pi =1}^{K}mP\left(\pi \right))$, and the remaining sort/filter stage is negligible for the small candidate portfolios used here. The dominant practical cost is therefore repeated world evaluation and robust candidate generation, which explains the large runtime difference relative to A* and scenario ensemble in Section 5.
**Algorithm 1** Conservative Robust Route Selection (CRRS)**Require:** Nominal world $W_{0}$, plausible worlds $W_{pl}$, start *s*, goal *g*, parameters
  1:Compute plausible-world distance transforms and robust clearance field $\rho_{\alpha_{g}}$  2:Build robust free set $F_{\rho }$ and robust clearance-weighted grid cost *G*  3:Generate heterogeneous candidate routes from robust-substrate, scenario, A*/Theta*, PRM, and visibility sources  4:Rasterize each route and compute nominal, plausible-world, and robust-substrate features  5:Apply dominance filtering using plausible collision and robust-substrate violation  6:Sort survivors lexicographically and select the best route using Equation (10) as a late tie-breaker  7:Apply guarded shortcut refinement only if the refined route does not worsen planning-time risk guards  8:**return** final path π


### 3.12. Interface to Downstream Tracking and Local Control

**Figure 2:**
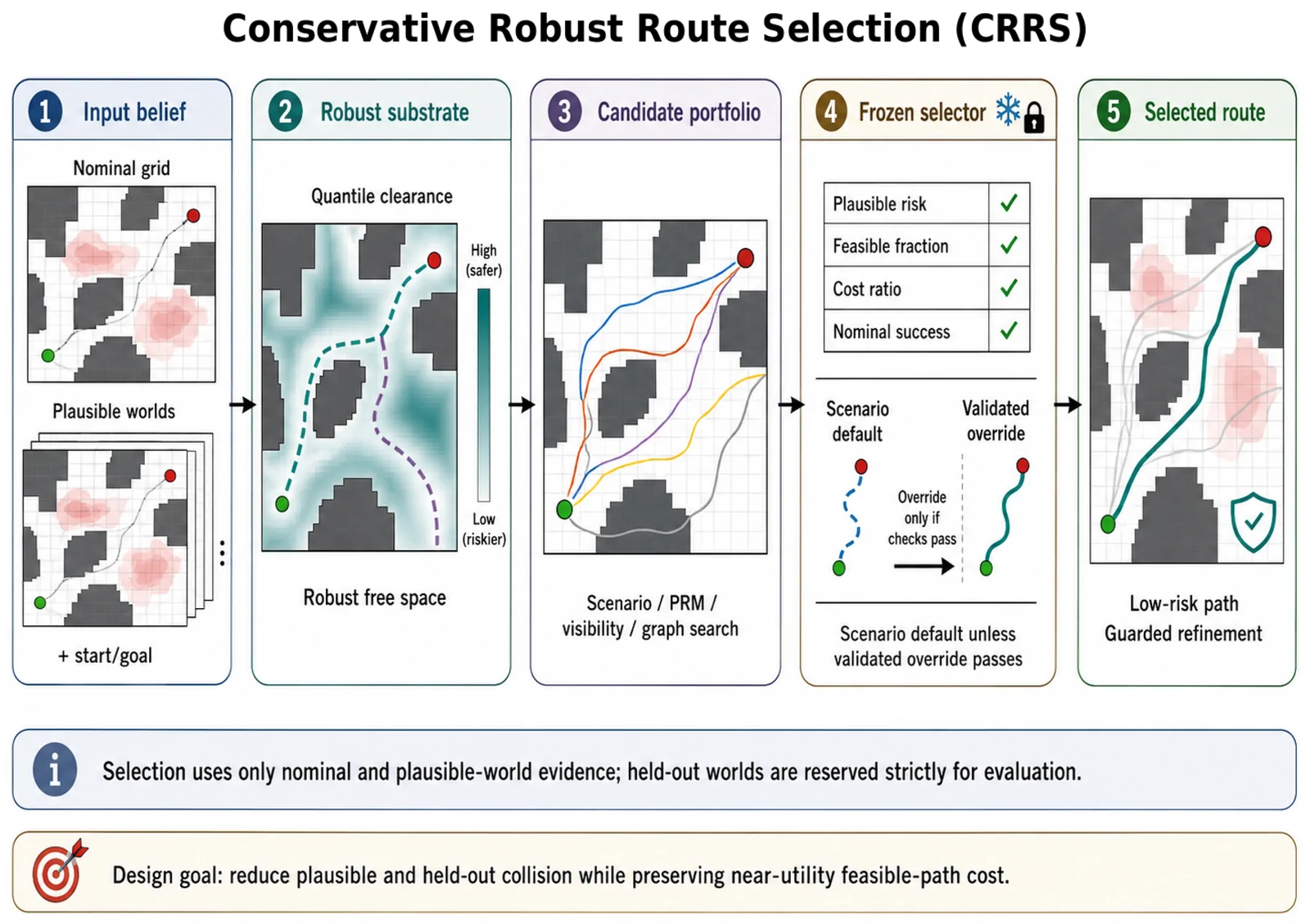
Sensor-uncertainty-aware Conservative Robust Route Selection (CRRS) pipeline for autonomous robotic path planning. Sensor-derived occupancy or traversability information defines a nominal map and a set of plausible occupancy-grid realizations representing occlusion, localization error, dynamic blockage, incomplete observation, and traversability uncertainty. CRRS constructs a robust clearance substrate, generates a heterogeneous candidate route portfolio, evaluates completed routes using nominal and plausible-world evidence only, applies a frozen conservative selector, and returns a selected route for downstream tracking or reactive control. Held-out worlds are reserved strictly for post-selection evaluation.

**Figure 3:**
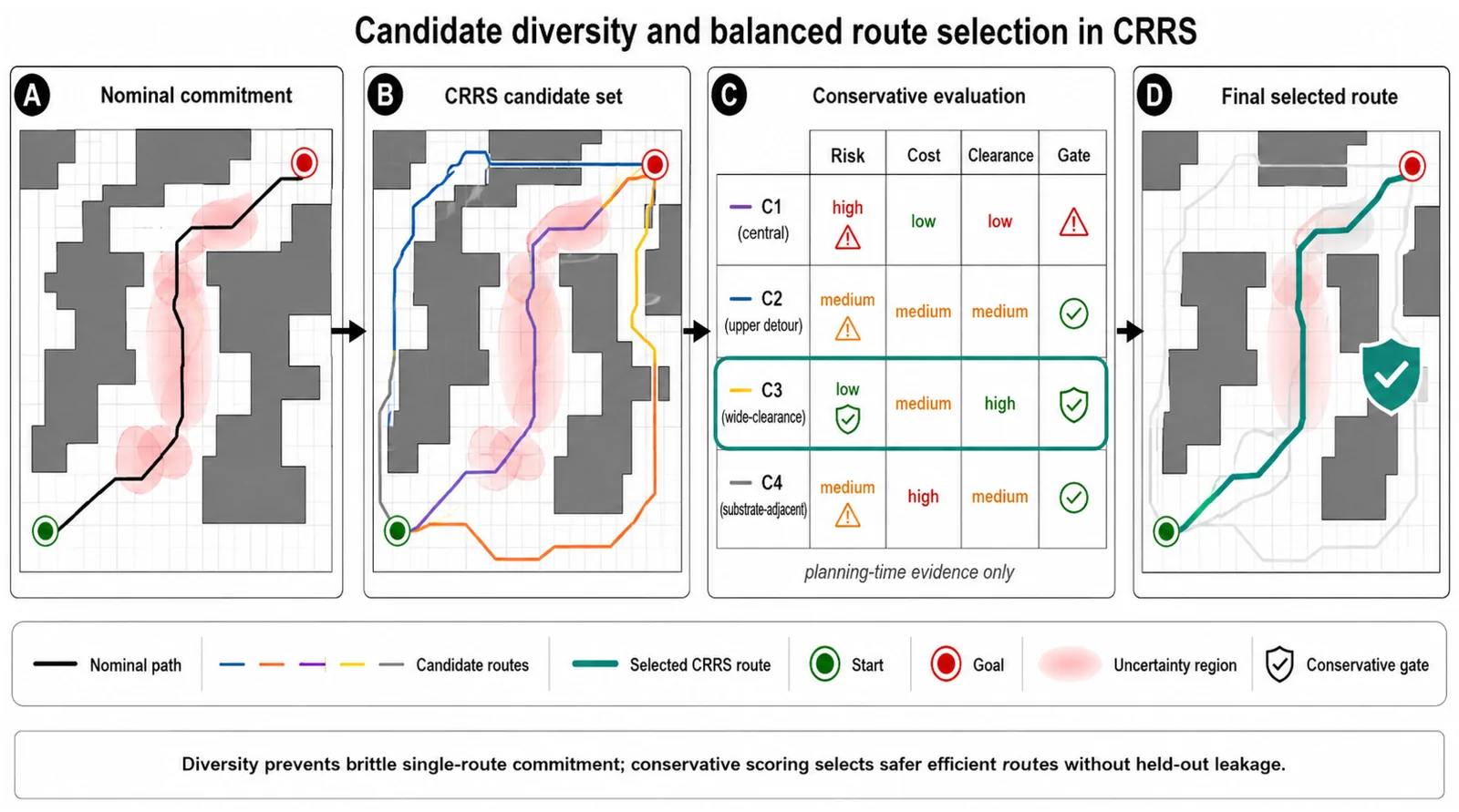
Candidate diversity and balanced route selection in CRRS. Candidate retention avoids premature commitment to a single brittle corridor by preserving alternatives from multiple route classes. The conservative evaluation stage selects a low-risk, efficient route using planning-time evidence over risk, cost, and clearance, without access to held-out outcomes.

CRRS returns a route-level object rather than a dynamically feasible closed-loop trajectory. The selected route can be expressed as a waypoint sequence

(14) $$\pi^{⋆}=\left(x_{1},x_{2},\ldots ,x_{n}\right),$$

or as a corridor around the selected route,

(15) $$C\left(\pi^{⋆},r\right)=\left\{x\in F:d\left(x,\pi^{⋆}\right)\leq r\right\},$$

where F is the nominal free space, $d(x,\pi^{⋆})$ is the distance from grid cell *x* to the selected route, and *r* is an application-dependent corridor radius. This interface allows CRRS to provide a topological route commitment, a waypoint reference, or a space corridor to a downstream module. A robot-specific planner or controller can then smooth the route using spline fitting, minimum-curvature smoothing, Hybrid A*, state-lattice planning, elastic-band methods, or trajectory optimization before execution. The smoothed trajectory may be passed to MPC, MPPI, DWA, TEB, pure-pursuit tracking, or another local controller. Consequently, the present evaluation measures route-level robustness before tracking and local collision avoidance; it does not claim that raw grid routes directly satisfy non-holonomic, acceleration, steering, or actuator constraints. Figure 2 summarizes the CRRS pipeline, and Figure 3 illustrates the retained candidate diversity and conservative route-selection stage.

## 4. Experimental Design

### 4.1. Evidence Path and Strict Superiority Gate

The evaluation uses a staged evidence path rather than a single tuned experiment. An initial implementation audit showed that direct heuristic overrides did not satisfy the strict comparison with the scenario ensemble. The evidence path is summarized in Table 5. The strict aggregate gate used for the final claim is

(16) $${\bar{p}}_{ho}\left(\pi_{method}\right)\leq {\bar{p}}_{ho}\left(\pi_{scenario}\right)\;\mathrm{and}\;{\bar{p}}_{pl}\left(\pi_{method}\right)<{\bar{p}}_{pl}\left(\pi_{scenario}\right),$$

where ${\bar{p}}_{pl}$ and ${\bar{p}}_{ho}$ denote the mean plausible and held-out collision rates, respectively, over matched domains and seeds.

### 4.2. Benchmarks, Splits, and Final Confirmation

The final confirmation uses a generated MovingAI-format benchmark with 30 map/ scenario domains, six families, and five seeds per domain group, yielding 150 matched groups. The families are chokepoints, maze-open, open-blocks, rooms, sparse-random, and warehouse. These are MovingAI-format generated fixtures rather than a claim over the official MovingAI benchmark corpus. The conservative override selector is trained and frozen before the final confirmation. The final confirmation then evaluates the frozen rule on the 30-domain benchmark without retuning.

The generated plausible and held-out worlds are used as controlled surrogates for uncertainty that would arise from a robot’s sensing and mapping pipeline. In a physical deployment, such uncertainty may originate from sensor occlusion, localization drift, map aging, temporary blockage, incomplete observation, or errors in traversability prediction. The benchmark worlds do not claim to reproduce a specific sensor model; instead, they provide repeatable occupancy-grid perturbations for testing whether route selection based on planning-time plausible worlds generalizes to disjoint held-out realizations. This interpretation keeps the evaluation aligned with sensor-derived robotic path planning while preserving a strict separation between planning information and post-selection assessment.

To strengthen external validity, we also evaluate the same selector on an official MovingAI long-distance subset derived from the public grid benchmarks of Sturtevant [25]. The subset contains 27 valid official map/scenario pairs across maze, random, room, street, and DAO families. One DAO map lacks queries satisfying the long-distance filter; the resulting run contains 260 start-goal problems, each with Euclidean start-goal distance of at least 100 grid cells. This public subset is used as an additional validation layer, not as a replacement for the frozen 30-domain confirmation.

We further add a LiDAR/SLAM-inspired simulated sensor-model validation on official MovingAI queries. This addendum uses structured occupancy-grid uncertainty that represents limited observed free-space, SLAM-like map perturbation, dynamic blockage blobs, plausible/held-out uncertainty worlds, and a risk field. It is included to strengthen the connection between the benchmark interface and sensor-derived map uncertainty, but it remains a simulated sensor-model validation rather than real LiDAR, camera, SLAM, ROS/Nav2/Gazebo, or hardware validation.

Finally, we add a controlled validation–held-out mismatch stress ablation. This ablation modifies a fixed subset of the official long-distance candidate records so that some candidates appear artificially attractive under validation and CVaR features while becoming brittle under held-out evaluation. The stress run is not the primary benchmark; it isolates whether the conservative CRRS gate adds value beyond a CVaR-only selector.

The final package also retains the earlier fair/stretch suites and smoke checks for reproducibility. Those older runs are useful for historical comparison and ablation context, but the main deployable claims are based on the frozen-selector confirmation, the official long-distance validation, the simulated sensor-model addendum, and the stress ablation described above.

### 4.3. Metrics and Statistical Reporting

A returned path is successful when it is collision-free in the nominal world. Throughout the manuscript, “plausible collision” denotes the mean rasterized route-collision rate across planning-time plausible worlds, whereas “held-out collision” denotes the corresponding mean rate across disjoint evaluation worlds. “Reference-grid collision” is used only in the simulated sensor-model addendum for collision on the unperturbed reference occupancy grid. These are grid-based route-evaluation metrics, not physical robot contact or real-world crash probabilities; accordingly, the term “true collision” is not used as a technical metric. Candidate generation and selector decisions may use nominal and plausible-world features only. Held-out worlds are used for evaluation and for oracle diagnostics, but never for CRRS inference.

We report paired deltas relative to the scenario ensemble. Negative deltas favor the proposed frozen selector. Confidence intervals are descriptive paired 95% intervals over matched groups or official-query instances, depending on the experiment. The generated-benchmark claim is aggregated over the 30-domain benchmark; the official MovingAI result is aggregated over 260 long-distance public-query instances. Family-level summaries are reported as diagnostics and limitations rather than as separate universal dominance claims. The paired confidence intervals are descriptive intervals over matched query or group records. Because queries drawn from the same map or family may be correlated, these intervals should not be interpreted as independent deployment-level guarantees; the family-level tables and win/loss/tie diagnostics are therefore reported alongside aggregate statistics, and hierarchical or cluster-bootstrap inference is left for future larger validation studies.

### 4.4. Reference-Baseline Diagnostic

The fair/stretch diagnostic suites were used to identify the appropriate primary comparator before the final confirmation study. Table 6 shows that the scenario ensemble was the strongest non-proposed baseline among the classical graph-search comparators: it reduced plausible and held-out collision relative to nominal A*, robust A*, and robust Theta* in both diagnostic regimes. Consequently, the final CRRS claim is intentionally evaluated against the scenario ensemble as the default action. This strengthens the evidence standard: CRRS must improve over a baseline that already captures sampled environment uncertainty, rather than merely outperforming deterministic single-route planners.

## 5. Results

The results are interpreted as route-level robustness evidence for autonomous robotic navigation over uncertain occupancy-grid maps. Plausible collision measures performance under planning-time sensor/map uncertainty, while held-out collision evaluates whether the selected route generalizes to disjoint environment realizations not used during route selection. These metrics therefore assess the robustness of the selected corridor before downstream path tracking, reactive obstacle avoidance, or low-level robot control. The reported improvements should be read as benchmark-level evidence for safer route commitment under sensor-derived map uncertainty, not as a claim of full closed-loop physical robot deployment.

### 5.1. Candidate-Pool Expansion and Oracle Headroom Study

The first empirical question was whether the available candidate pool contained any routes that could satisfy the strict criterion against the scenario ensemble. Table 7 reports the expanded raw-candidate audit. Across 150 final benchmark groups, the expansion study generated 65,550 raw candidate records and 17,343 unique candidates. Of these, 17,004 were successful unique candidates, and 1121 were strict-safe-superior relative to the scenario ensemble on their matched group. Strict-safe-superior candidates occurred in 113/150 groups.

Table 8 reports the oracle headroom summaries. The scenario-plus-safe oracle reduced plausible collision from 0.125 to 0.054 and held-out collision from 0.305 to 0.266. This result establishes strong headroom but is not a deployable result because the oracle uses held-out outcomes when choosing candidates.

The most productive candidate classes were hybrid scenario-clearance candidates, higher-scenario-count candidates, and PRM homotopy candidates (Table 9). This justifies the conservative override rule’s use of higher-scenario-count and PRM homotopy candidates as deployable override sources while retaining scenario ensemble as the default action.

### 5.2. Frozen-Selector Confirmation

Table 10 gives the frozen-selector confirmation result. Scenario ensemble has plausible collision of 0.1250 and held-out collision of 0.3053. The frozen CRRS conservative override selector has plausible collision of 0.0807 and held-out collision of 0.2937. The paired plausible-collision delta is −0.0443 with a 95% CI of [−0.0523, −0.0363]. The paired held-out delta is −0.0117 with a 95% CI of [−0.0207, −0.0026]. Thus the final frozen selector passes the predefined strict aggregate gate in Equation (16).

**Figure 4:**
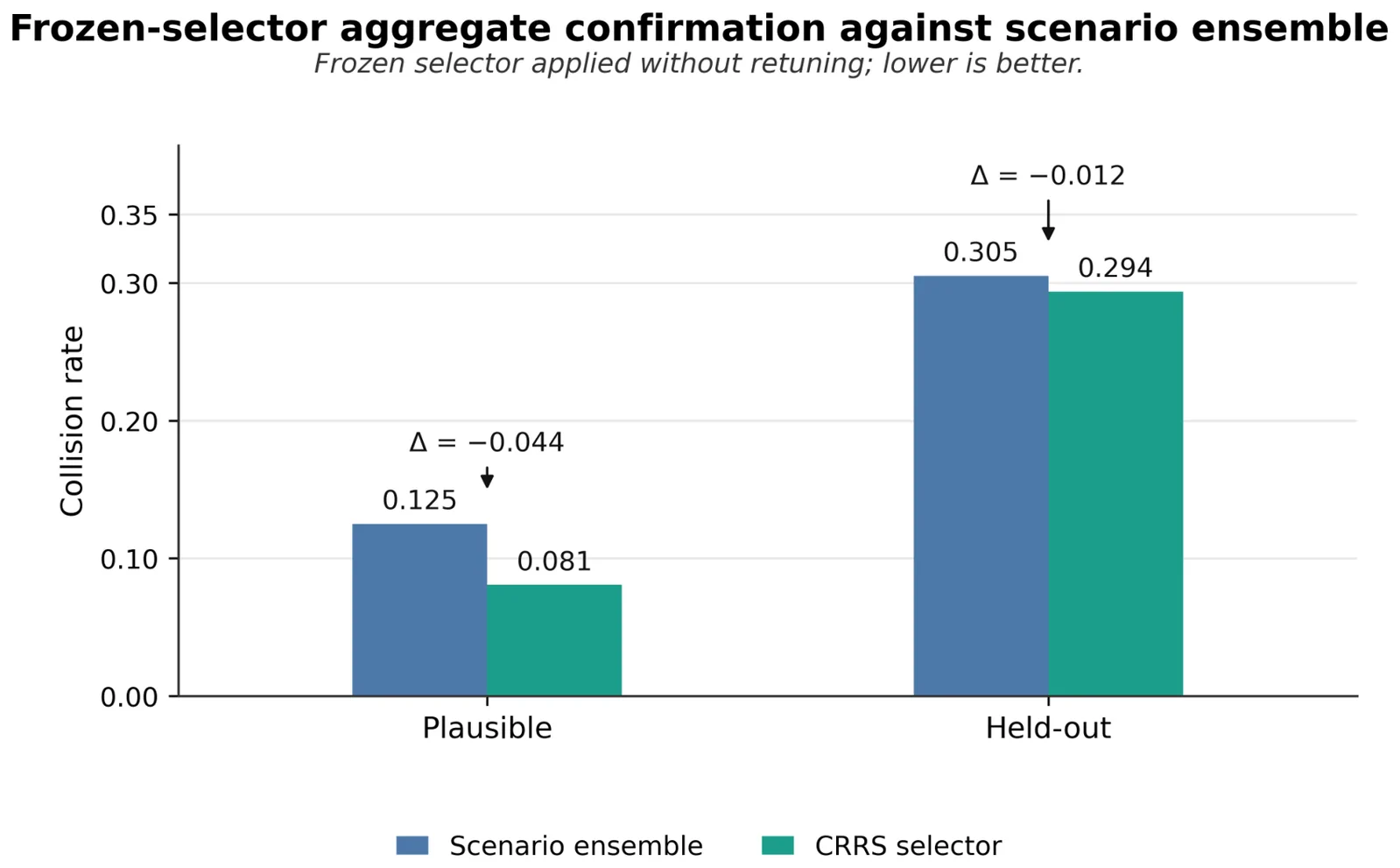
Frozen-selector aggregate confirmation against the scenario ensemble. On the 30-domain MovingAI-format benchmark, the frozen CRRS selector reduces plausible collision from 0.1250 to 0.0807 and held-out collision from 0.3053 to 0.2937 relative to the scenario ensemble. A lower collision rate is better. The selector is applied without retuning.

The aggregate frozen-selector comparison is visualized in Figure 4.

Table 11 records the final gate checks. The benchmark contains 150 groups, the frozen selector overrides the scenario ensemble in 88 groups, and 62 groups are strict-safe-superior at the group level. The final gate accepts the claim because the aggregate plausible improvement exceeds the required threshold, the held-out collision has a lower mean, and the held-out confidence interval remains within the predefined tolerance.

### 5.3. Family-Level Behavior

**Figure 5:**
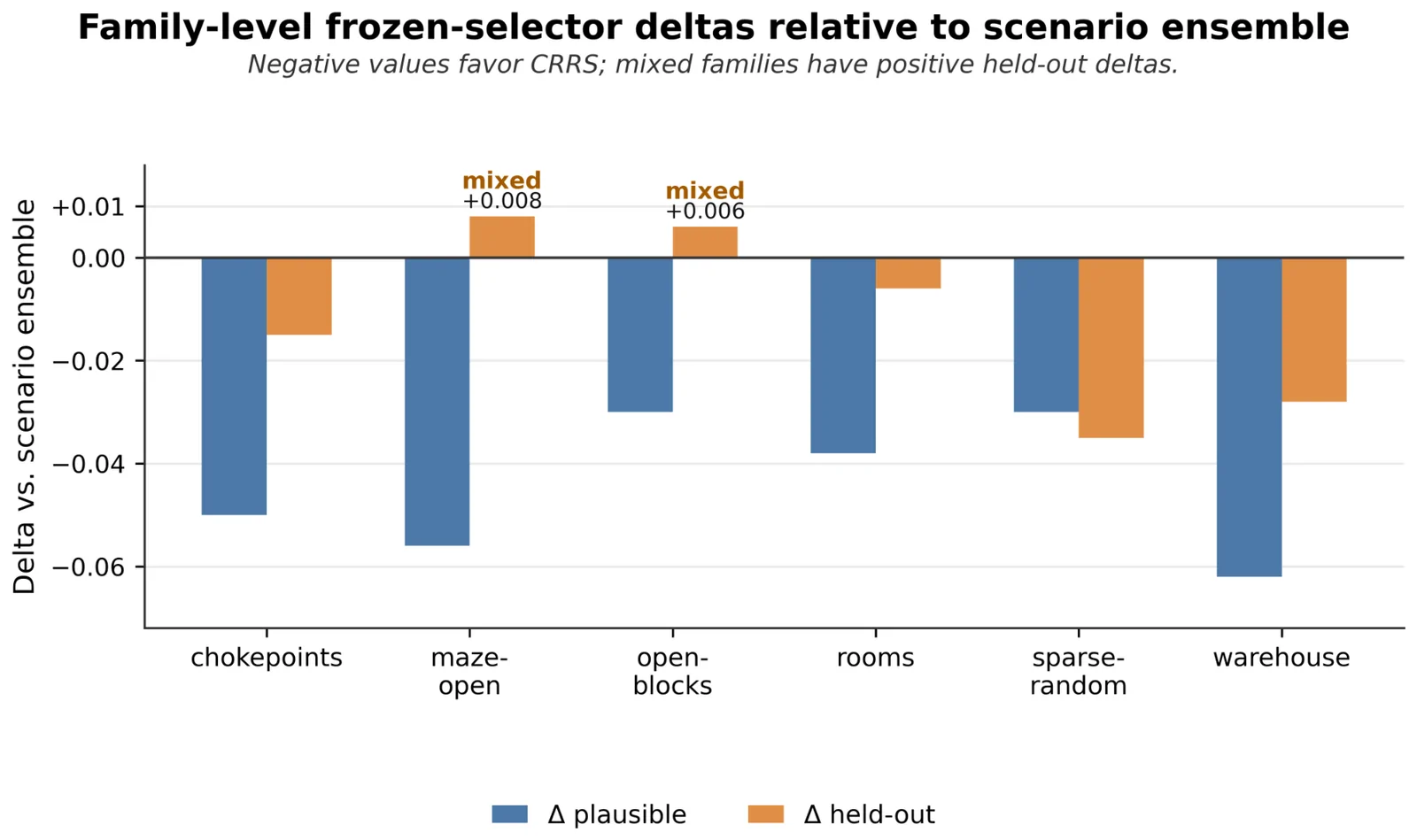
Family-level frozen-selector deltas relative to the scenario ensemble. Negative deltas favor the frozen CRRS selector. Chokepoints, rooms, sparse-random, and warehouse show favorable held-out deltas, whereas maze-open and open-blocks remain mixed because their held-out deltas are slightly positive despite lower plausible collision. The supported claim is aggregate benchmark superiority, not uniform per-family dominance.

Family-level analysis is shown in Table 12 and Figure 5. The aggregate benchmark result is favorable, but the result is not uniform across all families. Chokepoints, rooms, sparse-random, and warehouse have favorable mean held-out deltas. Maze-open and open-blocks have lower plausible collision but small positive held-out deltas. The added paired family columns make this boundary explicit: only sparse-random has a strictly negative held-out confidence interval in the generated benchmark, while chokepoints, rooms, and warehouse are favorable in mean but have family-level intervals that touch zero. Therefore, the supported claim is aggregate benchmark superiority, not uniform per-family dominance.

### 5.4. Topology-Aware Failure Boundary

The mixed family-level behavior defines an important failure boundary rather than a formatting detail. CRRS is most useful when there are meaningfully different route commitments: narrow chokepoints, separated corridors, or homotopy-like alternatives whose collision exposure differs across plausible worlds. It is less reliable when candidate routes have high geometric overlap, when open regions allow many visually different but risk-equivalent detours, or when a conservative detour increases path exposure without reducing held-out blockage risk. Table 13 summarizes this interpretation using practical topology proxies: chokepoint density, route redundancy, candidate route overlap, and expected detour exposure. These proxies explain why chokepoints, sparse-random, warehouse, and rooms are favorable in the generated benchmark, whereas maze-open and open-blocks are mixed. In the mixed families, lower plausible collision can fail to transfer because the plausible-world ensemble may favor longer detours whose additional exposure is not penalized enough under held-out perturbations. This motivates future selector variants that include explicit route-overlap, corridor-width, graph-connectivity, or homotopy-diversity penalties.

### 5.5. Official Long-Distance MovingAI Validation

Table 14 reports the official long-distance MovingAI validation. The run contains 260 public-query instances from 26 usable maps after filtering for start-goal distance of at least 100 cells. The mean start-goal distance is 298.5 cells, with a median of 255.7 and a maximum of 1244.0. Compared with the scenario ensemble, CRRS reduces plausible collision from 0.8159 to 0.6662 and held-out collision from 0.9648 to 0.8822. It also reduces mean path length from 839.6 to 792.9 cells while increasing runtime from 0.85 s to 4.74 s. These timings are not an equal-wall-clock or equal-collision-check budget comparison; they show that the reported robustness improvement is obtained at a higher computational cost. The absolute held-out collision values in this long-distance subset are grid-based risk scores under a deliberately severe uncertainty generator; they should not be interpreted as physical robot failure probabilities or closed-loop collision rates. The paired held-out delta is −0.0825 with a 95% CI of [−0.0976, −0.0682]. CRRS has 141 held-out wins, zero held-out losses, and 119 ties relative to the scenario ensemble. The same candidate records also support CVaR-only, risk-weighted, and compact *k*-shortest-style CVaR selectors, giving a same-pool risk-aware comparison. The *k*-shortest-style addendum is a penalty-diversified official-portfolio comparator rather than an exact Yen/Eppstein loopless enumeration; held-out quantities are used only for post-selection evaluation.

Family-level official results are shown in Table 15. The strongest gains occur on street and DAO maps, with moderate gains on maze maps and smaller but nonnegative mean gains on room and random maps. This pattern is consistent with the method’s purpose: CRRS is most useful when long-range route choice exposes substantial corridor-level risk differences.

**Figure 6:**
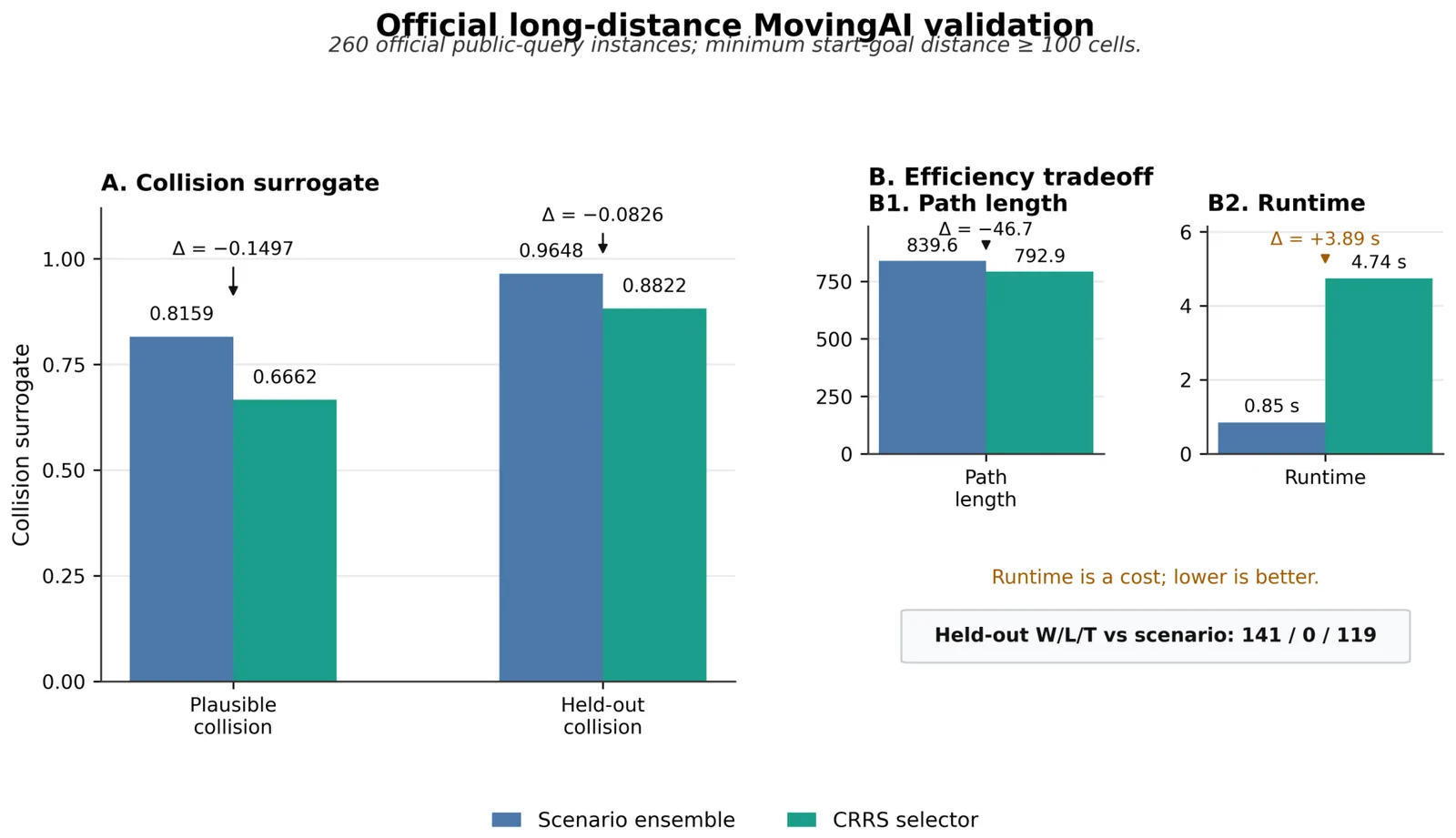
Official long-distance MovingAI validation. Over 260 official public-query instances with start-goal distance of at least 100 cells, CRRS reduces plausible collision, held-out collision, and path length relative to the scenario ensemble, while increasing runtime. The held-out comparison yields 141 wins, zero losses, and 119 ties for CRRS relative to the scenario ensemble. Lower is better for all plotted metrics.

Figure 6 summarizes the aggregate CRRS-versus-scenario official MovingAI collision, path-length, runtime, and held-out win/loss/tie results.

The official run also reveals an important ablation limitation. The CVaR-only selector, risk-weighted selector, and penalty-diversified *k*-shortest-style CVaR comparator are very strong on this benchmark and are close to CRRS. The *k*-shortest-style rows obtain a held-out collision value of 0.8800 compared with 0.8822 for CRRS, but the direct paired difference relative to CRRS is small, and its 95% confidence interval crosses zero (held-out delta −0.0023; 95% CI [−0.0063, 0.0011]; W/L/T 6/2/252). Thus, the official public benchmark supports the external validity of the broader risk-aware route-selection family over the scenario ensemble but by itself does not isolate the distinctive value of the conservative CRRS gate. The stress ablation below addresses that isolation question.

### 5.6. LiDAR/SLAM-Inspired Simulated Sensor-Model Validation

As an additional sensor-motivated validation layer, we added a structured simulated sensor-model addendum inspired by LiDAR/SLAM uncertainty on official MovingAI queries. The addendum constructs a nominal sensed occupancy grid and plausible/held-out uncertainty worlds using limited observed free-space coverage, SLAM-like map perturbation, dynamic blockage blobs, and a risk field. This experiment should be interpreted as simulated sensor-model validation at the occupancy-grid interface: it strengthens the connection to sensor-derived map uncertainty but does not constitute validation on real LiDAR, camera, SLAM log, ROS/Nav2/Gazebo execution, or physical robot data.

The structured run attempted the same 260 official long-distance queries and retained 200 routed problems; 60 problems were excluded because no collision-free route was available in the nominal sensed map. Table 16 reports the aggregate results. The table column labeled reference-grid collision denotes collision on the unperturbed reference occupancy grid used by the simulated sensor-model addendum; it is retained only as a sanity check that the selected routes remain feasible on the reference grid. It does not denote physical robot contact, closed-loop collision, or a real-world crash measurement. Relative to the scenario ensemble, CRRS reduces the held-out collision from 0.4059 to 0.3768, with a paired held-out delta of −0.0291 and a 95% CI of [−0.0401, −0.0189]. The direct held-out comparison gives 94 wins, 43 losses, and 63 ties for CRRS relative to the scenario ensemble. The CVaR-only and risk-weighted selectors also improve over the scenario ensemble, but CRRS obtains the lowest held-out collision and a shorter mean path than those risk-only selectors in this addendum.

A smaller sensor-smoke run is retained in the Supplementary Package as a diagnostic only. In that run, held-out collision differences were effectively neutral because the scenario-ensemble held-out collision was already very low; therefore, the manuscript emphasizes the structured addendum rather than the smoke diagnostic.

### 5.7. Validation–Held-Out Mismatch Stress Ablation

The stress ablation modifies 72/260 official long-distance problems by making selected low-CVaR candidates appear more attractive under validation while increasing their held-out brittleness and reducing clearance. This controlled manipulation tests whether a selector that optimizes only CVaR is vulnerable to harmful overrides and whether the CRRS abstention gate rejects those brittle choices.

Table 17 shows that CRRS remains favorable relative to the scenario ensemble under stress: held-out collision is reduced from 0.9648 to 0.8840, with a paired 95% CI of [−0.0957, −0.0663]. More importantly, CRRS separates from CVaR-only. CRRS differs from CVaR-only on 73/260 problems; in the held-out comparison, CRRS is better in 37 cases, CVaR-only is better in zero cases, and 223 cases tie. Relative to the scenario ensemble, CVaR-only incurs 18 harmful held-out losses, while CRRS incurs zero. This is the clearest evidence that the conservative override rule is not merely a CVaR selector: under validation–held-out mismatch, it prevents brittle low-CVaR overrides while preserving most of the scenario-ensemble improvement.

**Figure 7:**
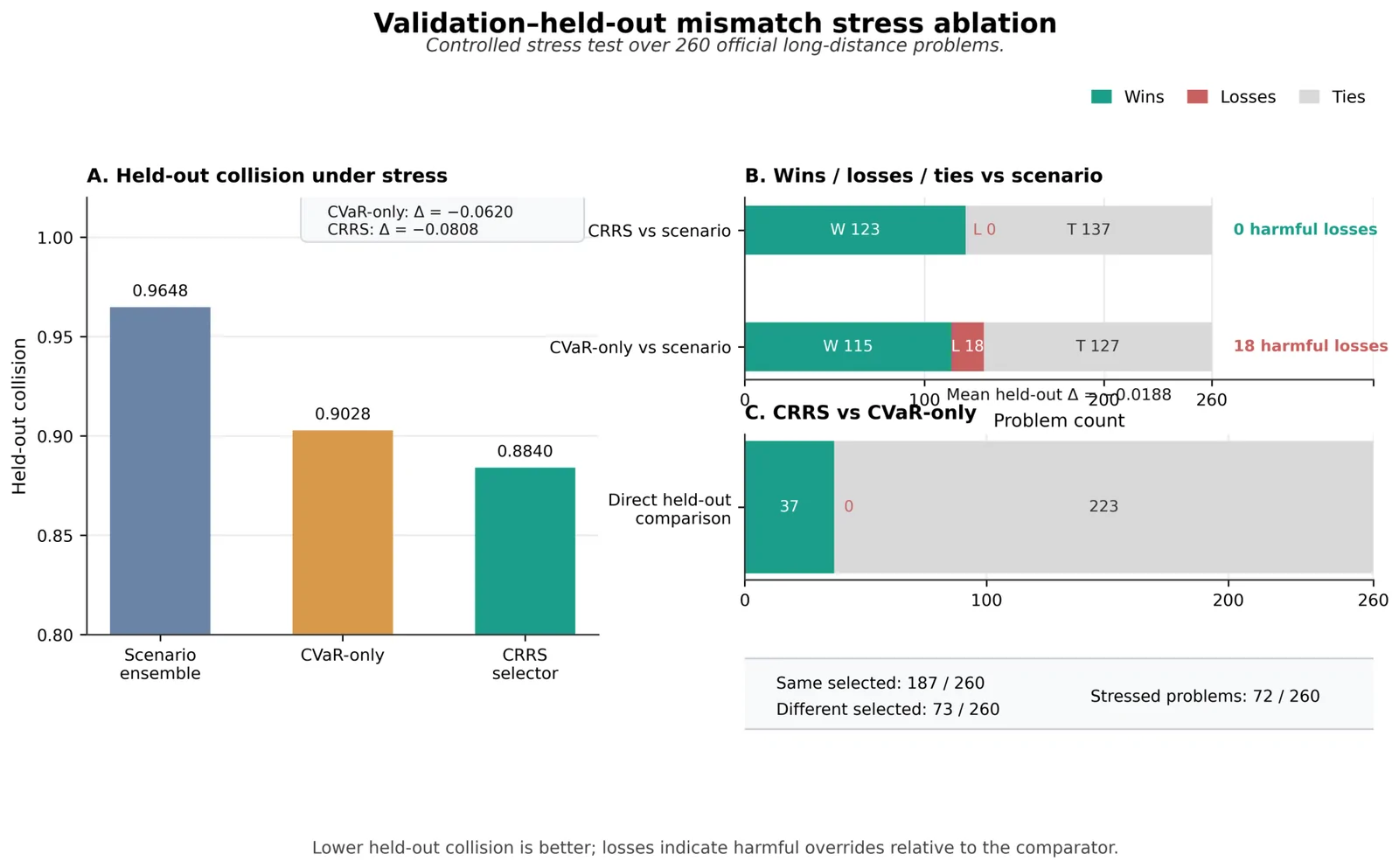
Validation–held-out mismatch stress ablation. Under controlled validation–held-out mismatch, CRRS preserves held-out improvement relative to the scenario ensemble while avoiding the harmful held-out losses incurred by CVaR-only selection. CVaR-only has 18 losses relative to the scenario ensemble, whereas CRRS has zero. In direct comparison, CRRS is better than CVaR-only in 37 cases, worse in zero, and tied in 223.

Figure 7 summarizes the stress-ablation held-out means, harmful-loss counts, and direct CRRS-versus-CVaR-only comparison.

### 5.8. Component Ablation and Selector Diagnostics

Component diagnostics were added to quantify which design choices matter most. Table 18 reports a controlled-domain ablation from the revision package. The ablation is not a full factorial proof, but it separates the main mechanisms: candidate-source availability, scalar-only selection, conservative guarding without a rich portfolio, and earlier aggressive selection. Removing the robust/portfolio-diversity source produces essentially the same plausible collision as the scenario ensemble and a slightly worse held-out collision, indicating that the candidate source is necessary. The scalar-only selector improves over the scenario ensemble but remains weaker than the full guarded selector. The guardians-only variant is fast but performs poorly because gating alone cannot compensate for a weak route portfolio. The earlier v4.2 full variant obtains a low plausible collision but a substantially worse held-out collision, illustrating why the final validation-frozen conservative selector is needed.

### 5.9. Parameter Sensitivity

Table 19 summarizes one-at-a-time sensitivity runs for the main quantile, tail-risk, and clearance parameters. Across the tested ranges, CRRS generally reduces plausible collision relative to the scenario ensemble, while held-out improvement varies with the setting. For example, $\alpha_{s}=0.40$ preserves the plausible-collision improvement but does not improve mean held-out collision in this suite, and β=0.75 yields only a small held-out reduction. These results support a conservative interpretation: the reported rule is not presented as universally optimal, and aggressive plausible-world tuning can fail to transfer to held-out worlds. This is why the final benchmark claim relies on the frozen selector and aggregate gate rather than post hoc parameter choice.

### 5.10. Computational Efficiency and Scalability

The computational cost reflects the deliberate use of finite-scenario evaluation and route portfolios. Table 20 reports the controlled-domain runtime audit. Classical graph search is orders of magnitude faster, the scenario ensemble is substantially faster than full CRRS, and the full balanced selector incurs the highest cost because it evaluates a richer route portfolio across plausible worlds. The no-robust-source and guardians-only ablations are faster but lose most of the robustness benefit, showing that runtime reduction by simply removing candidate diversity is not sufficient. On the official long-distance MovingAI validation, CRRS increased mean runtime from 0.848 s for the scenario ensemble to 4.740 s while reducing the held-out collision. This is a robustness-versus-computation tradeoff, not evidence of compute-normalized superiority. Therefore, CRRS should currently be interpreted as a deliberative global route-selection layer, not as a high-frequency reactive local planner.

All experiments were executed in a Python-based CPU environment on a local workstation with an Intel(R) Core(TM) i7-8565U CPU @ 1.80 GHz, four physical cores and eight logical threads, 32 GB RAM, and a 512 GB local fixed SSD drive. No GPU-specific implementation was used. The implementation used Python 3.12.13 with NumPy 2.3.5, pandas 3.0.1, SciPy, matplotlib, NetworkX, and image/grid-processing utilities from scikit-image and/or OpenCV where required. Unless otherwise stated, reported runtimes correspond to CPU execution on this platform. For a grid with *N* cells, *m* plausible worlds, *K* retained candidates, and P(π) raster samples along path π, robust distance-transform construction scales as O(mN) and candidate scoring scales as $O(\sum_{\pi =1}^{K}mP\left(\pi \right))$. Sorting and filtering are negligible for the small portfolios used here. The dominant practical cost is repeated world evaluation and robust candidate generation.

Runtime decomposition and acceleration path.

The runtime can be decomposed into plausible-world generation, distance-transform construction, candidate generation, candidate rasterization, candidate–world evaluation of collision and clearance, CVaR and robust-gap feature computation, and final sorting/gating. The candidate–world evaluation stage is largely decoupled: each pair of candidate route and plausible world can be evaluated independently once the worlds and rasterized paths are available. A practical acceleration path is therefore to batch route samples into arrays of size approximately K×m×P and compute collision indicators, clearance samples, CVaR features, and guard variables with vectorized CPU operations, multiprocessing, or GPU/CUDA kernels. This acceleration would not change the CRRS selector logic; it would only replace scalar loops over candidates and worlds with batched evaluation. The current implementation does not include such acceleration, so the reported method should be interpreted as an offline or high-level decision-support planner unless a parallel implementation is provided for a specific robot stack. Future work will investigate a PyTorch- or CuPy-based batched rasterization backend that moves the approximately K×m×P route–world evaluation operations to the GPU. This would enable evaluation of CRRS as an ROS 2/Nav2 global-route plugin on edge platforms such as NVIDIA Jetson, while preserving the same conservative selector semantics.

### 5.11. Role of Diagnostic Ablations

**Figure 8:**
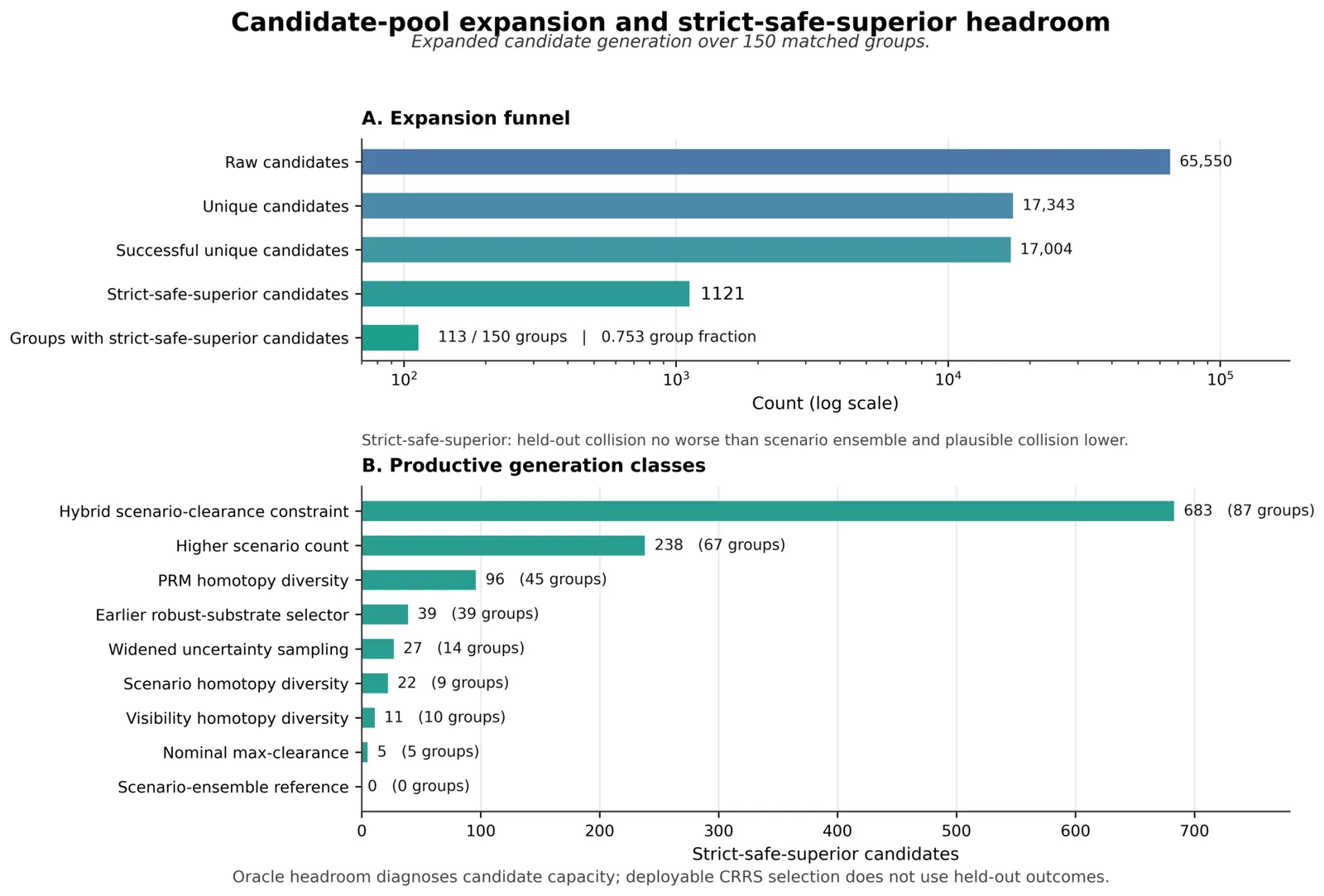
Candidate-pool expansion and strict-safe-superior headroom. Candidate-pool expansion produced 65,550 raw candidate records and 17,343 unique candidates across 150 matched groups. The expanded pool contains 17,004 successful unique candidates and 1121 strict-safe-superior candidates, with strict-safe-superior candidates appearing in 113/150 groups. Oracle headroom diagnoses candidate capacity only; deployable CRRS selection does not use held-out outcomes.

**Figure 9:**
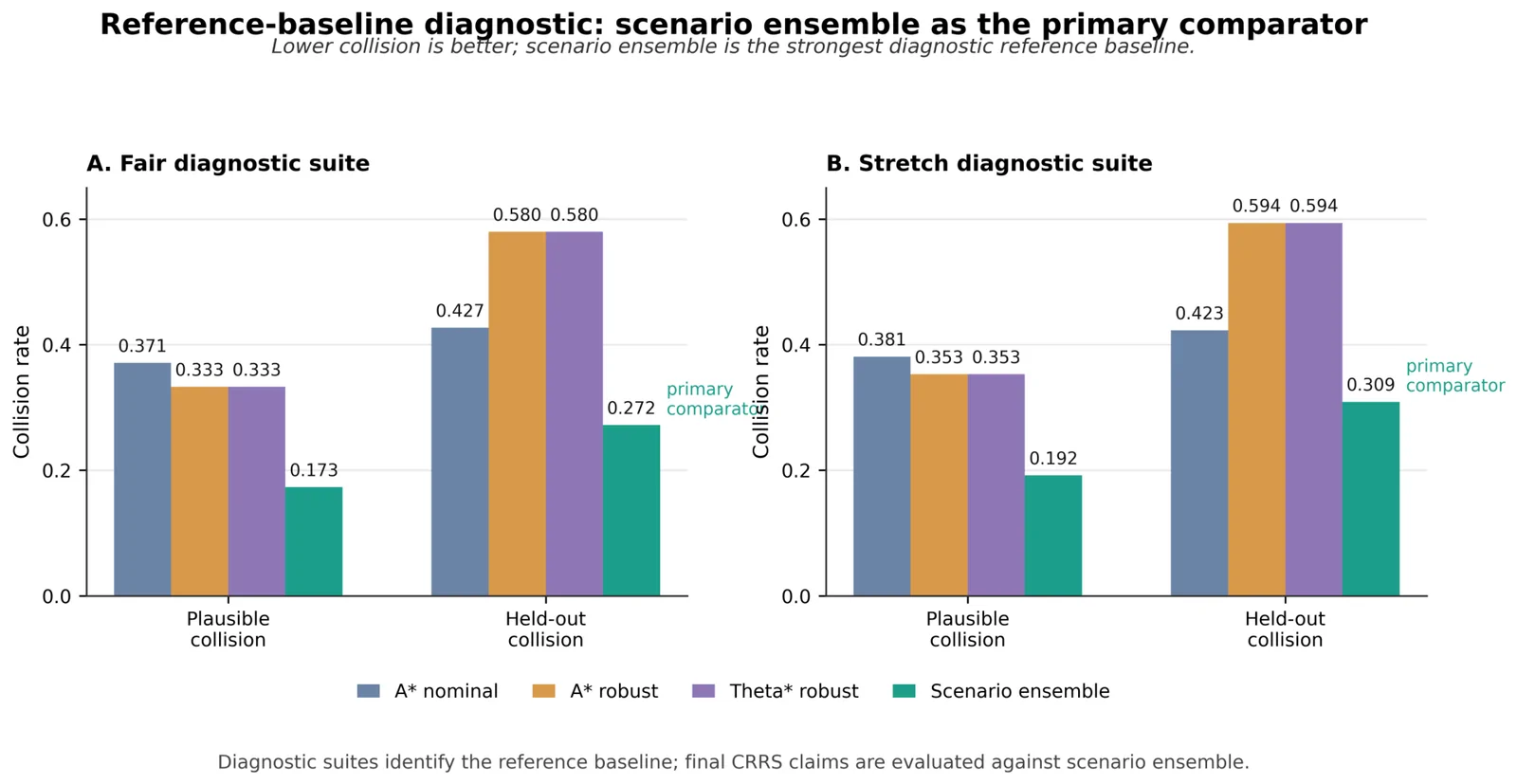
Reference-baseline diagnostic motivating scenario ensemble as the primary comparator. Scenario ensemble reduces plausible and held-out collision relative to nominal A*, robust A*, and robust Theta* in both fair and stretch diagnostic suites. This diagnostic establishes scenario ensemble as the strong default action and primary comparator for the final CRRS evaluation. Lower collision rate is better.

The fair/stretch diagnostic suites remain useful as ablations and reference-baseline evidence. They showed two important facts. First, single-route nominal and robust graph-search baselines were not the right primary comparison point under sampled uncertainty: scenario ensemble reduced held-out collision from 0.427 to 0.272 in the fair suite and from 0.423 to 0.309 in the stretch suite relative to nominal A*. It also improved substantially over robust A* and robust Theta*. Second, direct robust-substrate selection did not by itself provide a decisive deployable advantage over the scenario ensemble. The final claim therefore does not rely on prior internal versions. It relies on the professionalized evidence sequence used here: expanded raw-candidate headroom, validation-trained non-oracle selection, and frozen-rule final confirmation against the scenario ensemble. Figure 8 summarizes candidate-pool expansion and strict-safe-superior headroom, and Figure 9 summarizes the reference-baseline diagnostic that motivates scenario ensemble as the primary comparator.

## 6. Discussion

The final result clarifies the interpretation of the work. The diagnostic results show that the scenario ensemble is the meaningful reference baseline because it already dominates the A*/Theta* family under sampled uncertainty. This improves the novelty standard for CRRS: the method is not presented as a planner that merely beats deterministic shortest-path search but as an abstaining selector that can improve over a strong scenario-based default. The final CRRS evidence path addresses the remaining weakness by first testing candidate-pool headroom and then freezing a validation-trained selector before final confirmation. This is a stronger claim path because it separates three questions that were previously entangled: whether safe-superior candidates exist, whether a non-oracle selector can identify useful overrides, and whether the frozen rule generalizes to a larger benchmark.

From an optimization perspective, the main contribution is the separation between candidate generation, admissible-set construction, and frozen finite-scenario selection. The route is not chosen by a single nominal shortest-path objective; it is selected from a portfolio using collision, infeasibility, CVaR tail cost, robust-clearance, and cost-ratio information under a fixed data boundary. This framing is especially important for robotic path planning because it turns corridor choice under uncertainty into an auditable robust selection problem with explicit abstention.

The candidate-pool expansion study shows that candidate generation was the primary bottleneck. Selector-only tuning on the diagnostic pool produced sparse and non-generalizing improvements. By contrast, the expanded pool contains strict-safe-superior candidates in 113/150 final groups. The CRRS selector deliberately uses only a conservative subset of that pool. It defaults to the scenario ensemble and admits higher-scenario-count or PRM homotopy candidates only when planning-time plausibility, feasibility, and cost-ratio checks pass. This abstention mechanism is central to the method: the selector is not trying to replace the scenario ensemble in every case but to override it when the validation rule predicts a safer alternative.

The official long-distance MovingAI validation, simulated sensor-model addendum, and stress ablation strengthen but do not overextend this interpretation. The public MovingAI run shows that the CRRS-family selector and additional risk-aware portfolio comparators improve over the scenario ensemble on nontrivial official start-goal queries rather than only on generated MovingAI-format fixtures. The compact *k*-shortest-style addendum is especially useful as a strong comparator: it is nearly tied with CRRS on the ordinary official subset, which confirms that CRRS is being compared against nontrivial alternatives rather than weak deterministic paths. The LiDAR/SLAM-inspired addendum adds a more sensor-motivated occupancy-grid uncertainty model and shows a held-out reduction from 0.4059 to 0.3768 over 200 routed official queries. The stress ablation then shows why the conservative gate matters: when validation evidence underestimates brittle low-clearance candidates, CVaR-only selection can produce harmful overrides, whereas CRRS abstains or selects a safer candidate. This comparison also motivates a conservative reading of the *k*-shortest-style result: the penalty-diversified *k*-shortest-style CVaR comparator is statistically close to CRRS on the ordinary official subset, so the paper does not claim universal superiority over every route-portfolio selector. The distinct contribution is the conservative admissibility and abstention mechanism, which is most clearly isolated by the validation–held-out mismatch diagnostic.

Operational use in a robotic navigation stack.

In an autonomous navigation architecture, CRRS is intended to operate at the global route-selection layer, above trajectory tracking, low-level control, and reactive local collision avoidance. A perception, SLAM, learned traversability-estimation, prediction, or Digital Twin module would provide the nominal occupancy/traversability grid and the plausible-world set; CRRS would then select a route or corridor for downstream tracking, local planning, or controller execution. The conservative selector is designed to abstain when its admissibility guards fail, returning the scenario-ensemble route rather than forcing a risky override. This makes CRRS a robust route-commitment decision-support layer, not a replacement for local obstacle avoidance, trajectory tracking, actuator-level control, or online safety supervision.

Sim-to-real limitations.

The current evidence remains simulation- and benchmark-based. It does not validate physical sensor pipelines, ROS or other robot middleware integration, controller tracking error, computational or communication latency, localization-covariance propagation, closed-loop replanning under moving dynamic agents, robot dynamics and actuation limits, Digital Twin validation, or hardware trials. CRRS is nevertheless compatible with Digital Twin and hardware-in-the-loop validation because its interface consumes nominal and plausible occupancy or traversability worlds. A calibrated Digital Twin could generate physically grounded plausible worlds from robot dynamics, sensor models, and environment updates, but such validation was not performed in this study. Future work should test CRRS in a Digital Twin or hardware-in-the-loop setup before ROS-based mobile-robot deployment with live LiDAR, stereo, or sonar perception.

The result is still bounded. The generated confirmation is not a proof over all maps, and the official MovingAI subset covers selected long-distance grid queries rather than a full deployment distribution. Two generated-benchmark families show small positive held-out deltas despite favorable aggregate performance. Runtime remains higher than lightweight graph-search and scenario-only planners because the method evaluates a large candidate pool, and the present experiments do not establish equal-budget superiority under a fixed wall-clock or collision-check budget. These limitations do not negate the final aggregate result, but they define its appropriate scope.

## 7. Limitations and Reproducibility

The experiments are grid-based and simulation-based. The final 30-domain benchmark is MovingAI-format generated data, and the official MovingAI validation is a selected long-distance subset rather than the entire official corpus. The added LiDAR/SLAM-inspired validation uses a simulated structured sensor model at the occupancy-grid interface; it is not real LiDAR, camera, SLAM-log, ROS/Nav2/Gazebo, or physical robot validation. The uncertainty model is finite and sampled; it is not a formal distributional guarantee over all possible deployment conditions. Runtime comparisons are implementation- and hardware-dependent, and the evidence supports robustness at higher computational cost rather than compute-normalized superiority. The family-level analysis shows that the method is not uniformly dominant: maze-open and open-blocks families have lower plausible collision but small positive held-out deltas in the generated benchmark, and gains for the random and room families are small in the official long-distance run. The stress ablation is a controlled diagnostic designed to expose selector behavior under mismatch; it should not be interpreted as a natural benchmark distribution. Likewise, the LiDAR/SLAM-inspired addendum is a structured simulated sensor-model study at the occupancy-grid interface; it strengthens sensor grounding but does not replace calibrated sensor logs, middleware-in-the-loop tests, or physical robot experiments.

Reproducibility is a central part of the contribution. The revised submission is organized around separate evidence archives: the C4 generated-benchmark final package reproduces the 30-domain frozen-selector confirmation; the official MovingAI package contains the 260-query public-benchmark candidate records, selected-route outputs, paired statistics, win/loss/tie summaries, anytime/runtime summaries, and stress-ablation scripts; the sensor-validation addendum contains the LiDAR/SLAM-inspired structured candidate records, selected routes, paired statistics, failure list, and code-diff patch; and the controlled MovingAI output package contains the component-ablation, sensitivity, runtime, and candidate-budget diagnostics. The Nav2/Gazebo materials, where included, are preparation and replay assets only; no live simulator or physical robot deployment claim is made from them. Additional reproducibility files and supporting results are provided in the Supplementary Materials. Appendix A provides the mapping between publication-facing terminology and archived artifact names.

## 8. Conclusions

This paper presents Conservative Robust Route Selection (CRRS), a finite-scenario robust multi-candidate route-selection framework for path planning under sampled environment uncertainty with a frozen conservative override selector and a strict evaluation boundary. Diagnostic evidence shows why the scenario ensemble is the appropriate primary comparator: it substantially outperforms nominal A*, robust A*, and robust Theta* under the fair/stretch uncertainty suites. Against that stronger reference, the final confirmation result supports a benchmark-scoped superiority claim on the 30-domain MovingAI-format generated benchmark: plausible collision is reduced from 0.1250 to 0.0807 and held-out collision from 0.3053 to 0.2937, with favorable paired confidence intervals for both deltas. The official long-distance MovingAI validation further reduces the held-out collision from 0.9648 to 0.8822 over 260 public queries, with no held-out losses relative to the scenario ensemble. The LiDAR/SLAM-inspired simulated sensor-model addendum further reduces the held-out collision from 0.4059 to 0.3768 over 200 routed official queries, while remaining clearly distinct from real-sensor or hardware validation. Finally, the validation–held-out mismatch stress ablation isolates the conservative gate: CRRS avoids the 18 harmful held-out losses incurred by CVaR-only selection and is never worse than CVaR-only in held-out comparison. The added *k*-shortest-style comparator is therefore interpreted as a strong official-benchmark addendum rather than as changing the paper’s main scoped claim.

The main limitations are deliberately scoped. The present study evaluates CRRS in grid-based simulation and benchmark settings rather than on a physical robot with live sensor streams. The plausible and held-out worlds approximate uncertainty that can arise in sensing and mapping pipelines, but they do not constitute a calibrated model of any specific LiDAR, vision, sonar, or multi-sensor perception system. Runtime is also higher than lightweight graph-search and scenario-ensemble baselines; thus, the method should be treated as a deliberative robustness layer unless a compute-budget-matched implementation is provided. The family-level results show that the aggregate advantage is not uniform across all map types. Although this study does not perform Digital Twin validation, the CRRS interface is compatible with Digital Twin and hardware-in-the-loop pipelines because it consumes nominal and plausible occupancy or traversability worlds. A calibrated Digital Twin could provide physically informed realizations derived from robot dynamics, sensor models, and environment updates. Future work should therefore integrate CRRS with ROS-based navigation stacks, sensor-derived occupancy or traversability maps, dynamic-obstacle updates, Digital Twin validation, and hardware-in-the-loop testing before claiming full closed-loop robotic deployment.

## Figures and Tables

**Table 1:** Scope of the present evidence versus future robotic deployment validation.

Aspect	Demonstrated in This Paper	Not Claimed/Future Work
Route-level robustness	Generated MovingAI-format confirmation, official MovingAI validation, stress ablation, and LiDAR/SLAM-inspired simulated sensor-model addendum	Distribution-free safety guarantee over all deployment conditions
Sensor uncertainty	Controlled occupancy-grid perturbations and structured LiDAR/SLAM-inspired simulated plausible/held-out worlds	Calibrated physical LiDAR, camera, sonar, or SLAM-log validation
Robot execution	Route/corridor interface for downstream smoothing, local planning, and tracking	Closed-loop ROS/Nav2/Gazebo, Webots, Isaac Sim, or physical robot deployment
Runtime	CPU runtime audit, complexity, scalability discussion, and acceleration path	Real-time superiority over local planners or high-frequency reactive control
Family behavior	Aggregate benchmark improvement with family-level diagnostics	Uniform dominance across all map families

**Table 2:** Terminology and information-boundary definitions used in CRRS.

Term	Meaning in This Paper
Nominal world $W_{0}$	The occupancy or traversability grid available to the robot at planning time.
Plausible worlds $W_{pl}$	Planning-time alternative occupancy/traversability realizations used for candidate generation, scoring, filtering, and the deployable selector.
Held-out worlds $W_{ho}$	Evaluation-only realizations used after route selection; no held-out metric is used by the deployable CRRS selector.
Plausible collision	Mean rasterized route-collision rate across the planning-time plausible worlds $W_{pl}$.
Held-out collision	Mean rasterized route-collision rate across the evaluation-only held-out worlds $W_{ho}$; it is not used by the deployable selector and is not a physical crash probability.
Reference-grid collision	Collision on the unperturbed reference occupancy grid in the simulated sensor-model addendum; this is a feasibility sanity check, not physical robot contact.
Scenario ensemble	The conservative scenario-based default route and primary comparator. CRRS returns this route whenever no admissible override is accepted.
Candidate portfolio	The set of completed route candidates generated before final selection from scenario, robust-substrate, graph-search, PRM/homotopy, visibility-style, or other allowed sources.
CRRS selector	The frozen non-oracle conservative override rule evaluated using nominal and plausible-world features only.
CVaR-only selector	A risk-aware comparator that selects by plausible-world tail-risk evidence without the conservative CRRS abstention gate.
Risk-weighted selector	A comparator using a weighted risk score over the same candidate records.
Oracle/headroom selector	A diagnostic selector allowed to inspect held-out outcomes to measure candidate-pool capacity; it is not deployable and does not support the final non-oracle claim.

**Table 3:** Interpretation of benchmark uncertainty surrogates as sensor-derived map uncertainty. The present experiments test the route-selection interface, not a calibrated physical sensor simulator.

Benchmark Surrogate	Robotic Interpretation	Example Sensor or Mapping Source
Obstacle insertion or local blockage patches	Temporary blockage, newly detected obstacle, or short-lived dynamic obstruction in the local map	LiDAR, RGB-D, sonar, or local occupancy-grid updates from moving agents or clutter
Obstacle dilation or conservative inflation	Localization uncertainty, safety inflation, or pose-covariance margin around occupied cells	SLAM covariance, odometry drift, map-registration uncertainty, or inflated cost-map layers
Clearance degradation or robust-clearance quantile shift	Reduced traversability confidence and uncertainty in obstacle boundary location	Vision/LiDAR terrain classifier confidence, stereo-depth uncertainty, noisy range returns, or learned traversability scores
Corridor closure or blocked passage	Occlusion, incomplete observation, or missed free-space evidence in a narrow passage	Limited field of view, blind zones, map staleness, sensor occlusion, or unobserved rooms/corridors
Validation–held-out mismatch	Perception or deployment shift between planning-time evidence and future realizations	Lighting/weather change, dynamic clutter, unmodelled occlusion, map aging, or changing obstacle occupancy

**Table 4:** Terms in the CRRS base route-selection score. Negative weights reward clearance.

Term	Definition	Weight
${\hat{p}}_{pl}\left(\pi \right)$	Mean plausible collision rate	85.000
$b_{pl}\left(\pi \right)$	Fraction of plausible worlds with infeasible-penalty cost	20.000
$v_{\rho }\left(\pi \right)$	Fraction of path samples below $\rho_{min}$ on robust substrate	2.000
$\Delta_{q10}\left(\pi \right)$	$\max(\rho_{min}-q_{0.10}^{\rho }\left(\pi \right),0)$	0.750
$o_{\rho }\left(\pi \right)$	Fraction of path samples outside robust free set	0.400
${\mathrm{CVaR}}_{\beta }\left(C_{i}\left(\pi \right)\right)$	Plausible-world tail cost	0.003
${\bar{C}}_{pl}\left(\pi \right)$	Mean plausible-world cost	0.001
$C_{0}\left(\pi \right)$	Nominal-world cost	0.012
$d_{0}^{min}\left(\pi \right)$	Nominal minimum clearance	−0.060
$\bar{\rho }\left(\pi \right)$	Mean robust-substrate clearance	−0.035
${\bar{d}}_{pl}\left(\pi \right)$	Mean plausible-world minimum clearance	−0.015

**Table 5:** Evaluation evidence path. Candidate-pool expansion establishes headroom; validation-based selector design fixes a non-oracle rule; frozen-selector confirmation performs the final benchmark evaluation.

Stage	Role	Outcome
Implementation audit	Verification and repair	Exact scenario-reference behavior matched the scenario ensemble; direct heuristic overrides remained insufficient for a strict claim.
Candidate-pool expansion	Candidate-generation expansion and oracle headroom test	65,550 raw and 17,343 unique candidates over 150 groups; strict-safe-superior candidates appeared in 113/150 groups.
Validation-based selector design	Non-oracle selector trained on validation families	The frozen rule selected higher-scenario-count and PRM homotopy candidates while abstaining to the scenario ensemble otherwise.
Frozen-selector confirmation	Frozen-rule MovingAI-format 30-domain confirmation	Passed the strict aggregate gate: plausible collision 0.0807 vs. 0.1250 and held-out collision 0.2937 vs. 0.3053.
LiDAR/SLAM-inspired addendum	Simulated sensor-model validation on official queries	On 200 routed problems, CRRS reduced held-out collision from 0.4059 to 0.3768; this is simulated sensor-model evidence, not real-sensor or hardware validation.

**Table 6:** Diagnostic reference-baseline comparison. Lower collision is better. Scenario ensemble dominates nominal A*, robust A*, and robust Theta* on the fair/stretch diagnostic suites, motivating its role as the default action and primary comparator in the final confirmation study.

Planner	Plausible_*fair*_	Held-Out_*fair*_	Plausible_*stretch*_	Held-Out_*stretch*_
A* nominal	0.371	0.427	0.381	0.423
A* robust	0.333	0.580	0.353	0.594
Theta* robust	0.333	0.580	0.353	0.594
Scenario ensemble	**0.173**	**0.272**	**0.192**	**0.309**

*Note: Bold values indicate the lowest collision rate in each column.*

**Table 7:** Candidate-pool expansion raw-candidate quality on the 30-domain MovingAI-format benchmark. Strict-safe-superior means held-out collision no worse than the scenario ensemble and plausible collision lower than the scenario ensemble for the same matched group.

Quantity	Value
Domains	30
Groups	150
Raw candidate records	65,550
Unique candidate records	17,343
Unique successful candidates	17,004
Unique strict-safe-superior candidates	1121
Groups with strict-safe-superior candidates	113
Strict-safe-superior group fraction	0.753
Raw success rate	0.912
Unique success rate	0.980

**Table 8:** Oracle headroom summaries on the final 30-domain benchmark. Negative deltas favor the oracle relative to the scenario ensemble. These rows diagnose headroom and do not themselves authorize a deployable superiority claim.

Oracle	Plausible	Held-Out	Δ Plausible	Δ Held-Out
heldout_oracle	0.148	0.212	0.023	−0.093
constrained_superiority_oracle	0.065	0.255	−0.060	−0.050
scenario_plus_safe_oracle	0.054	0.266	−0.071	−0.040
source_oracle	0.054	0.266	−0.071	−0.040

**Table 9:** Candidate quality by generation class. The final frozen CRRS rule uses a conservative subset of these sources rather than the full oracle pool.

Generation Class	Unique	Success	Strict-Safe	Groups
Hybrid scenario-clearance constraint	4350	4224	683	87
Higher scenario count	4390	4362	238	67
PRM homotopy diversity	4956	4889	96	45
Earlier robust-substrate selector	150	150	39	39
Widened uncertainty sampling	320	320	27	14
Scenario homotopy diversity	1514	1413	22	9
Visibility homotopy diversity	721	719	11	10
Nominal max-clearance	792	777	5	5
Scenario-ensemble reference	150	150	0	0

**Table 10:** Frozen-selector MovingAI-format 30-domain confirmation. The frozen rule is applied without retuning. Negative deltas favor the frozen selector.

Method	Plausible	Held-Out	Δ Plausible	Δ Held-Out
Scenario ensemble	0.125	0.305	–	–
CRRS selector	0.081	0.294	−0.044	−0.012
Paired 95% CI	–	–	[−0.052, −0.036]	[−0.021, −0.003]

**Table 11:** Final confirmation gate checks. These gates were defined before interpreting the final MovingAI-format 30-domain result.

Quantity	Value	Gate
Groups	150	≥100
Override count	88	–
Override rate	0.587	≥0.10
Strict-safe-superior groups	62	–
Strict-safe-superior group fraction	0.413	≥0.20
Minimum plausible improvement	0.044	≥0.015
Held-out delta	−0.012	≤0
Held-out CI95 upper bound	−0.003	≤0.010

**Table 12:** Family-level paired deltas in the frozen-selector confirmation. Negative deltas favor the frozen selector. The held-out 95% CI and W/L/T columns are paired diagnostics; families marked mixed have lower plausible collision but small positive held-out deltas.

Family	Groups	Δ Plausible	Δ Held-Out	Held-Out 95% CI	Held-Out W/L/T	Overrides	Strict-Safe Groups	Family Gate
Chokepoints	25	−0.050	−0.015	[−0.035, 0.005]	11/3/11	19	16	Pass
Maze-open	25	−0.056	0.008	[−0.007, 0.023]	4/5/16	10	5	Mixed
Open-blocks	25	−0.030	0.006	[−0.007, 0.019]	4/7/14	13	6	Mixed
Rooms	25	−0.038	−0.006	[−0.014, 0.002]	6/3/16	13	10	Pass
Sparse-random	25	−0.030	−0.035	[−0.067, −0.003]	9/3/13	12	9	Pass
Warehouse	25	−0.062	−0.028	[−0.058, 0.002]	14/5/6	21	16	Pass

**Table 13:** Topology-aware interpretation of generated-benchmark family behavior. The categories are diagnostic proxies derived from the observed family behavior and route-selection mechanism; they are not claimed as a full topological invariant computation.

Family	Observed Held-Out Transfer	Dominant Topology Proxy	Interpretation
Chokepoints	Favorable	High bottleneck contrast, meaningful corridor alternatives	Conservative selection avoids brittle bottlenecks when plausible worlds reveal blockage risk.
Rooms	Favorable	Doorway-mediated route classes, moderate redundancy	Route alternatives differ enough for scenario evidence to select safer commitments.
Sparse-random	Favorable	Irregular obstacles, moderate route diversity	Candidate diversity creates useful detours without excessive route-overlap penalty.
Warehouse	Favorable	Structured aisles and separated corridors	Alternative aisles provide meaningful robust route choices under blockage uncertainty.
Maze-open	Mixed	High overlap among many locally similar routes	Plausible-world gains can overfit local perturbations while detours increase held-out exposure.
Open-blocks	Mixed	Open-space detours, weak chokepoint structure	Longer conservative routes may add exposure without reducing the held-out collision rate.

**Table 14:** Official long-distance MovingAI validation with same-pool risk-aware and compact *k*-shortest-style comparators. Lower is better for collision, path length, and runtime. Method rows report deltas relative to the scenario ensemble; the final row reports the direct *k*-shortest-style versus CRRS comparison.

Method	Plausible	Held-Out	Path Length	Runtime (s)	Held-Out W/L/T	Δ Held-Out
Scenario ensemble	0.8159	0.9648	839.6	0.85	–	–
CVaR-only	0.6680	0.8832	792.9	4.74	141/0/119	−0.0815
Risk-weighted ensemble	0.6680	0.8832	792.9	4.74	141/0/119	−0.0815
CRRS selector	0.6662	0.8822	792.9	4.74	141/0/119	−0.0825
Nominal *k*-shortest-style + CVaR	0.6580	0.8800	795.4	4.70	143/1/116	−0.0848
Robust *k*-shortest-style + CVaR	0.6580	0.8800	795.4	4.70	143/1/116	−0.0848
Paired 95% CI for CRRS delta	[−0.1767, −0.1228]	[−0.0976, −0.0682]	[−57.74, −36.34]	[2.50, 5.50]	–	–
*k*-shortest-style vs. CRRS delta	[−0.0161, −0.0024]	[−0.0063, 0.0011]	[0.14, 6.05]	[−0.120, 0.001]	6/2/252	−0.0023

**Table 15:** Official long-distance MovingAI family-level held-out deltas. Negative deltas favor CRRS relative to scenario ensemble.

Family	Problems	Wins	Losses	Ties	Mean Held-Out Delta
street	50	46	0	4	−0.1938
dao	50	42	0	8	−0.1418
maze	60	17	0	43	−0.0646
room	30	15	0	15	−0.0128
random	70	21	0	49	−0.0059

**Table 16:** LiDAR/SLAM-inspired simulated sensor-model validation on official MovingAI queries. The addendum reports 200 routed problems out of 260 attempted; 60 were excluded because no collision-free route was available on the nominal sensed map. Lower is better for collision, path length, and runtime. This is simulated sensor-model validation, not real LiDAR/SLAM or hardware validation.

Method	Plausible	Held-Out	Reference-Grid Collision	Path Length	Runtime (s)	Held-Out W/L/T	Δ Held-Out
Scenario ensemble	0.4063	0.4059	0.0000	515.1	1.040	–	–
CVaR-only	0.2180	0.3853	0.0000	535.8	1.389	89/45/66	−0.0206
Risk-weighted ensemble	0.2060	0.3847	0.0000	536.4	1.370	90/50/60	−0.0212
CRRS selector	0.2278	0.3768	0.0000	519.8	1.303	94/43/63	−0.0291
Paired 95% CI for CRRS held-out delta	–	[−0.0401, −0.0189]	–	–	–	–	–

**Table 17:** Validation–held-out mismatch stress ablation. The top block compares CRRS and CVaR-only against the scenario ensemble; the bottom block isolates CRRS versus CVaR-only.

Comparison	Held-Out Mean	Δ Held-Out	95% CI	Wins	Losses	Ties
CRRS vs. scenario ensemble	0.8840	−0.0808	[−0.0957, −0.0663]	123	0	137
CVaR-only vs. scenario ensemble	0.9028	−0.0620	[−0.0762, −0.0483]	115	18	127
Risk-weighted vs. scenario ensemble	0.9028	−0.0620	[−0.0762, −0.0483]	115	18	127
CRRS vs. CVaR-only	–	−0.0188	–	37	0	223
Same selected candidate	187/260					
Different selected candidate	73/260					
Stressed problems	72/260					

**Table 18:** Component-style ablation on the controlled-domain revision suite. Lower collision is better. Runtime and candidate counts are reported when available. The table is diagnostic rather than a full factorial decomposition.

Variant	Diagnostic Role	Plausible	Held-Out	Runtime (s)	Cand.
Scenario ensemble	Default and primary comparator	0.1886	0.2880	2.44	11.0
CRRS v5.1 balanced hybrid	Full guarded portfolio selector	0.1122	0.2642	32.05	18.0
No robust/portfolio-diversity source	Tests robust/portfolio-diversity source contribution	0.1886	0.2890	4.05	17.0
Scalar-only selector	Tests score-only selection without full hierarchy	0.1592	0.2817	31.79	18.0
Guardians only	Tests guards without full route portfolio	0.2780	0.3240	1.42	5.0
Earlier aggressive CRRS variant	Earlier aggressive selector diagnostic	0.1142	0.3692	27.87	4.5

**Table 19:** One-at-a-time sensitivity audit. “pl.” denotes plausible collision and “ho.” denotes held-out collision. Deltas are CRRS minus scenario ensemble; negative values favor CRRS.

Setting	Scenario pl.	CRRS pl.	Δ pl.	Scenario ho.	Δ ho.
$\alpha_{g}=0.02$	0.1448	0.0954	−0.0495	0.2757	−0.0200
$\alpha_{g}=0.04$	0.1639	0.0954	−0.0685	0.2657	−0.0267
$\alpha_{g}=0.08$	0.1734	0.0954	−0.0780	0.2824	−0.0167
$\alpha_{s}=0.20$	0.1639	0.0754	−0.0885	0.2657	−0.0281
$\alpha_{s}=0.30$	0.1639	0.0954	−0.0685	0.2657	−0.0267
$\alpha_{s}=0.40$	0.1639	0.0954	−0.0685	0.2657	0.0000
β=0.75	0.1639	0.0884	−0.0755	0.2657	−0.0052
β=0.85	0.1639	0.0954	−0.0685	0.2657	−0.0267
β=0.95	0.1639	0.0752	−0.0886	0.2657	−0.0433
$\rho_{min}=1.50$	0.1639	0.0889	−0.0749	0.2690	−0.0067
$\rho_{min}=1.85$	0.1639	0.0954	−0.0685	0.2657	−0.0267
$\rho_{min}=2.20$	0.1591	0.1258	−0.0333	0.2490	−0.0267

**Table 20:** Runtime audit on the controlled-domain revision suite. P90 is the 90th percentile runtime. Mean candidate count is reported for portfolio-based planners. Runtime values correspond to CPU execution on the local workstation described in the text.

Planner/Variant	Mean (s)	Median (s)	P90 (s)	Mean Cand.
A* nominal	0.022	0.019	0.042	–
A* robust	0.028	0.021	0.046	–
Theta* robust	0.349	0.232	0.747	–
Scenario ensemble	2.437	2.271	3.708	11.0
CRRS v5.1 balanced hybrid	32.048	26.145	62.646	18.0
No robust/portfolio-diversity source	4.051	3.611	6.686	17.0
Guardians only	1.424	1.302	2.651	5.0

## Data Availability

The revised Supplementary Package provides the result tables and scripts supporting the generated 30-domain frozen-selector confirmation, official MovingAI long-distance validation, compact *k*-shortest-style addendum, LiDAR/SLAM-inspired simulated sensor-model validation addendum, validation–held-out mismatch stress ablation, component-ablation diagnostics, sensitivity analyses, and runtime audits. Oracle/headroom outputs are used only for candidate-pool analysis and are not deployable selector evidence. The Supplementary Package also documents the CPU-based execution environment used for the reported runs.

## Supplementary Materials

The following supporting information can be downloaded at https://www.mdpi.com/article/10.3390/s26165172/s1. The revised supplementary package includes the generated 30-domain final-confirmation archive, the official MovingAI validation/stress-ablation archive, the compact *k*-shortest-style addendum, the LiDAR/SLAM-inspired simulated sensor-model validation addendum, the controlled ablation/sensitivity/runtime archive, frozen selector configuration files, verification scripts, and reproducibility notes. Nav2/Gazebo files are provided only as replay/preparation assets unless separate executed simulator logs are supplied.

## Appendix A. Reproducibility Artifact Name Mapping

The paper uses publication-facing terminology for the method and evidence path. Some released filenames retain internal provenance labels so that the reported results can be traced exactly to the archived experiment packages. The mapping is as follows: Implementation Integrity Audit corresponds to the B3.2 audit package; Candidate-Pool Expansion and Oracle Headroom Study corresponds to the C2-B package; Validation-Based Selector Design corresponds to the C3 package and frozen selector rule; and Frozen-Selector Confirmation corresponds to the C4 final confirmation package. These labels are retained only for repository traceability and are not separate methods.
